# Performance of computational algorithms to deconvolve heterogeneous bulk ovarian tumor tissue depends on experimental factors

**DOI:** 10.1186/s13059-023-03077-7

**Published:** 2023-10-20

**Authors:** Ariel A. Hippen, Dalia K. Omran, Lukas M. Weber, Euihye Jung, Ronny Drapkin, Jennifer A. Doherty, Stephanie C. Hicks, Casey S. Greene

**Affiliations:** 1 Department of Systems Pharmacology and Translational Therapeutics, University of Pennsylvania, Philadelphia, PA USA; 2 Penn Ovarian Cancer Research Center, Department of Obstetrics and Gynecology, Perelman School of Medicine, University of Pennsylvania, Philadelphia, PA USA; 3 Department of Biostatistics, Johns Hopkins Bloomberg School of Public Health, Baltimore, MD USA; 4 Huntsman Cancer Institute, University of Utah, Salt Lake City, UT USA; 5 Department of Biomedical Informatics, University of Colorado Anschutz Medical Campus, Aurora, CO USA

**Keywords:** Deconvolution, Tumor composition, Single-cell RNA-seq

## Abstract

**Background:**

Single-cell gene expression profiling provides unique opportunities to understand tumor heterogeneity and the tumor microenvironment. Because of cost and feasibility, profiling bulk tumors remains the primary population-scale analytical strategy. Many algorithms can deconvolve these tumors using single-cell profiles to infer their composition. While experimental choices do not change the true underlying composition of the tumor, they can affect the measurements produced by the assay.

**Results:**

We generated a dataset of high-grade serous ovarian tumors with paired expression profiles from using multiple strategies to examine the extent to which experimental factors impact the results of downstream tumor deconvolution methods. We find that pooling samples for single-cell sequencing and subsequent demultiplexing has a minimal effect. We identify dissociation-induced differences that affect cell composition, leading to changes that may compromise the assumptions underlying some deconvolution algorithms. We also observe differences across mRNA enrichment methods that introduce additional discrepancies between the two data types. We also find that experimental factors change cell composition estimates and that the impact differs by method.

**Conclusions:**

Previous benchmarks of deconvolution methods have largely ignored experimental factors. We find that methods vary in their robustness to experimental factors. We provide recommendations for methods developers seeking to produce the next generation of deconvolution approaches and for scientists designing experiments using deconvolution to study tumor heterogeneity.

**Supplementary Information:**

The online version contains supplementary material available at 10.1186/s13059-023-03077-7.

## Background

Solid tumors are highly heterogeneous tissues; the malignant cancer cells cohabitate and interact with various immune and stromal cells, known broadly as the tumor microenvironment (TME), in complex ways [1]. For cancer patients with the same tumor type, differences in the TME can yield different outcomes in progression, treatment response, and overall survival. TME composition affects immune cells’ ability to locate and kill malignant cells, the bioavailability and effectiveness of chemotherapy drugs, the availability of oxygen and other nutrients needed for cancer cell growth, and the possibility of metastasis [2, 3]. For these reasons, thorough characterization of the TME is an active area of cancer research [4, 5].

Researchers often use bulk RNA sequencing (RNA-seq) and single-cell RNA-seq (scRNA-seq) to examine the TME. Bulk sequencing—extracting RNA from pulverized tissue—is cost-effective and allows for transcriptome-wide coverage of total RNA. Many large cancer characterization efforts, such as The Cancer Genome Atlas, have bulk RNA-sequenced hundreds or thousands of samples [6]. Unfortunately, bulk RNA-seq loses direct information on tumor purity and cell type composition. Single-cell RNA-seq involves dissociating tissue and characterizing individual cells, retaining cell type-specific information. However, scRNA-seq is expensive and thus hard to scale to large datasets. scRNA-seq also produces much sparser data than bulk RNA-seq [7]. Each data modality presents unique experimental opportunities and challenges, but it is possible to combine bulk and single-cell data to computationally estimate tissue composition of bulk RNA-seq data using single-cell profiles, providing estimates of the TME for larger studies.

In the context of the TME, deconvolution describes the challenge of estimating cell type abundances from bulk profiles. Methods can be reference-free [8–10] or reference-based [11–15]. Many reference-based methods use a matrix of signature marker genes, but with the advent of single-cell sequencing, reference-based methods using profiles drawn from single-cell observations have become widespread. We focus on reference-based methods in this paper.

Whether or not methods use single-cell data as input, many within-method validations and cross-method benchmarks rely on single-cell data to assess the accuracy of a deconvolution method [16–18]. These assessments aggregate scRNA-seq data to create simulated or “pseudo-bulk” tumors with known cell type proportions. This assumes that single-cell and aggregated bulk data are biologically equivalent and that performing well on one data type indicates capturing similar information on the other. However, there are several technical differences that strain this assumption.

One source of technical variability between single-cell and bulk sequencing is dissociation. Separating cells from each other requires vigorous chemical and/or physical digestion, which can lyse cell membranes or otherwise compromise cell integrity [19]. Certain cell types are more sensitive to this process and are systematically underrepresented in scRNA-seq data [20]. Deconvolution algorithms that assume complete representation of cell types may perform well on pseudo-bulk assessments but could underperform in practice.

Another difference between single-cell and bulk RNA sequencing is the method of mRNA enrichment. Most RNA in any given cell is ribosomal RNA, which is undesired in most RNA-seq studies [21]. There are two prevailing ways to enrich for non-ribosomal RNA [22]. Many bulk RNA-seq experiments use ribosomal depletion which directly removes rRNA from a sample. This approach performs well for capturing partially-degraded RNA, such as that found in formalin-fixed paraffin-embedded (FFPE) tissue [23]. An alternative strategy is poly-A capture, which adds primers that ligate to the polyadenylated 3′ ends of mRNA. Many single-cell protocols use poly-A-based methods. It is unknown how using reference profiles from poly-A captured single cells affects the deconvolution of rRNA-depleted bulk samples.

More obstacles arise for scientists looking to perform deconvolution on tumor data. While some deconvolution methods are designed for cancer data [11, 17], comparative benchmarks have been performed predominantly on normal tissue [16, 18]. Solid tumors present unique challenges in deconvolution. Aberrant and dysregulated tissue growth often yields incomplete dissociation with many cells damaged [24]. Inter-patient heterogeneity is also much greater for malignant cells than for normal cell types [25], making it harder to generalize patterns across samples. Indeed, robustness to the noise contributed by the tumor fraction has been called one of the major challenges deconvolution algorithms face [26], highlighting the need to consider cancer data when comparing utility of deconvolution methods.

In this work, we generate a unique dataset of high-grade serous ovarian tumors and use it to directly examine the effects of protocol differences and their ramifications for deconvolution. We evaluate the feasibility of generating a reference profile from scRNA-seq samples that have been pooled across multiple tumors and compare hash and genetic demultiplexing as ways to reconstruct sample of origin information. We compare gene expression from dissociated and non-dissociated tissue from the same tumors to assess how dissociation affects cell type representation. We also perform differential expression of matched rRNA-depleted and poly-A captured tissues to see how different mRNA enrichment methods affect the expression profile. We then compare the consistency of six deconvolution methods across protocols and assess their accuracy on cancer data. Finally, we propose a series of recommendations for researchers looking to sequence cancer samples for use in deconvolution and subsequent at-scale studies of the TME.

## Results

### Experimental design

Our dataset comprises tumor data from *n *= 8 high-grade serous ovarian carcinoma (HGSOC) patients. HGSOC is known to have considerable inter-patient and intra-tumor heterogeneity, making deconvolution particularly valuable [27–29]. In addition, HGSOC tumors exemplify the kinds of challenges faced in cancer sequencing. Since HGSOC easily disseminates through the peritoneal cavity and forms small metastases, most debulking surgeries are extensive and take many hours [30], increasing the RNA and tissue degradation prior to freezing or fixture. The tumor’s histopathology is marked by extensive regions of necrotic tissue [31] resulting in a large amount of cellular debris at sequencing. Also, HGSOC cells have high genomic instability and a particularly high burden of copy number variants [32, 33]. CNVs can complicate deconvolution by altering the baseline of gene expression in cancer cells in a tumor-specific manner, therefore increasing inter-patient heterogeneity. By focusing on a challenging tumor type, we aim to identify best practices that are robust to real-world experimental conditions and thus have relevance to many other solid tumor types.

**Fig. 1:**
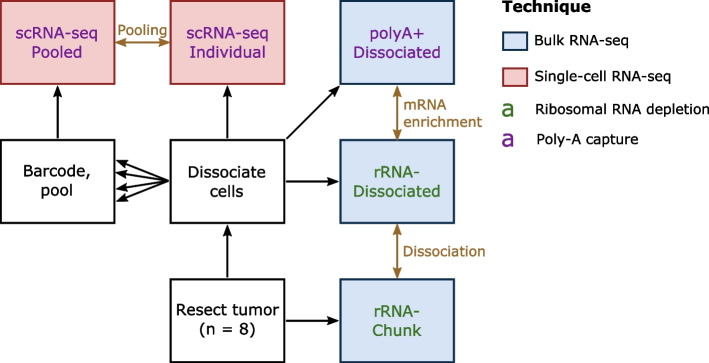
Overview of experimental design. Each tumor was profiled in five different ways, three times with bulk RNA-seq (blue box) and twice with scRNA-seq (red box) using two strategies for mRNA enrichment, rRNA depletion (green text) and poly-A capture (purple text). Gold text represent analyses that performed prior to deconvolution, with gold arrows signifying the datasets that compared in that analysis. Note that every data type will be used for the comparison of deconvolution methods

We used data from eight HGSOC tumors for which frozen tumor chunks and frozen dissociated cells were available (Methods). To directly assess the ways different library preparation methods affect deconvolution in cancer data, we assayed our data in multiple ways (Fig. 1). We performed RNA extraction on the tumor chunks, enriched for mRNA with rRNA depletion, and performed bulk RNA-sequencing. We will refer to this data type as “rRNA^-^ Chunk.” Ribo-depletion on undigested tissue is one of the most common protocols for cancer RNA-seq datasets and is thus likely to be used as an input for deconvolution. We also performed rRNA depletion on dissociated cells and performed bulk RNA-sequencing. We will refer to this data type as “rRNA^-^ Dissociated.” By comparing the rRNA^-^ Chunk and rRNA^-^ Dissociated data, we examine the effect of dissociation without other confounding factors that would be involved in bulk vs. single-cell comparisons. We also performed poly-A (3′) capture and performed bulk RNA-sequencing on RNA from dissociated cells. We will call this data type “polyA^+^ Dissociated.”

In addition to our three bulk sequencing data types, we performed two different scRNA-seq assay types. For one portion of the dissociated cells, we performed scRNA-seq on each tumor separately. We will refer to these as the “scRNA-seq Individual” samples. For another portion of the dissociated cells, we added a barcoded antibody and pooled the cells into batches (two sets of four samples each) and performed scRNA-seq on the pools. We will refer to these as the “scRNA-seq Pooled” samples. Performing scRNA-seq both individually and in pools allows us to directly compare deconvolution results using reference profiles from each data type and also evaluate the impact of demultiplexing on deconvolution.

### Multiplexing increases scRNA-seq throughput while preserving sample-specific information

Pooling has the potential to greatly increase the scalability of single-cell profiling, but it introduces technical and computational challenges. Pooled samples require a higher total number of cells to be loaded for acceptable coverage of each sample. In cancer samples with high cellular debris from necrotic tissue, loading more cells may increase the risk of clogging the microfluidic device. By splitting our samples into two pools of four and adding an extra debris-filtering step on the batched samples (Methods), we could sequence cell counts comparable to or higher than the individual single-cell runs (Table 1).

**Table 1:** Single-cell count per sample. All numbers are after filtering based on percentage of mitochondrial reads

Sample ID	Sample type	Cells	Component samples
2251	Individual	9464	
2267	Individual	5345	
2283	Individual	7627	
2293	Individual	10609	
2380	Individual	6300	
2428	Individual	283	
2467	Individual	6729	
2497	Individual	9313	
A	Pooled	7358	2267, 2283, 2293, 2380
B	Pooled	9814	2251, 2428, 2467, 2497

Upon successful sequencing, another challenge arises: identifying from which sample each cell originates. The process of computationally splitting the cells into groups by sample or patient of origin is known as demultiplexing. To determine if it is possible to sufficiently demultiplex cells from cancer tissue to use them as reference profiles for deconvolution, we performed two kinds of demultiplexing: hash demultiplexing and genetic demultiplexing.

#### Hash demultiplexing of pooled data is precise but limited at default thresholds

**Fig. 2:**
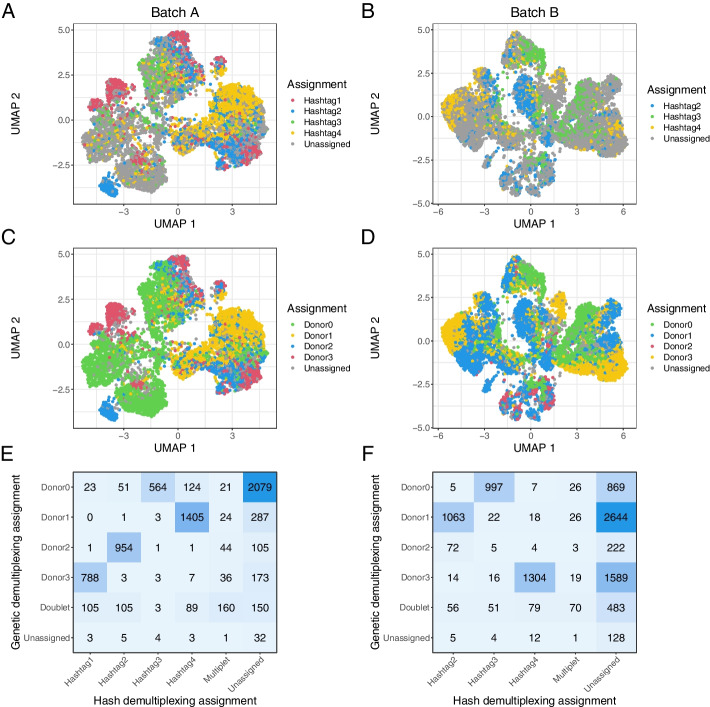
Results of antibody-based and genetic demultiplexing are concordant in cancer data. **A**, **B** A UMAP representation of the pooled data from batch A (**A**) and batch B (**B**), colored by antibody-based assignment from cellranger multi. **C**, **D** The same samples colored based on genetic demultiplexing assignment from vireo. **E**, **F** A confusion matrix showing the overlap of assignments with antibody-based and genetic demultiplexing in each sample

For hash demultiplexing, cells are labeled with an antibody targeting ubiquitous cell surface epitopes attached to a unique oligo-tag (one for each sample) and are then pooled; after sequencing, the tag on each cell is used to recapitulate the sample of origin [34]. We used 10X Genomics’ cellranger multi platform to do this. When performing demultiplexing based on antibody hashing in the two batches, 4246 and 3734 of the cells respectively (57.7% and 38.0%) were assigned to one sample, with 286 and 145 (3.9% and 1.5%) cells called as multiplets and 2823 and 5935 (38.4% and 60.5%) cells unassigned (Fig. 2A, B, Additional file 1: Tables S1–S2).

When reviewing the assignment probabilities for each cell, we found that many unassigned cells mapped to one antibody hashtag with reasonably high probability. The default assignment threshold for cellranger multi is 90% probability or greater of originating from one sample. When we relaxed this threshold to 85% probability, 4974 and 3831 cells were assigned (67.6% and 39.0% of total) (Additional file 2: Fig. S1A-B). A further relaxed threshold of 80% probability yielded 5536 and 4032 assigned cells (75.2% and 41.1% of total) (Additional file 2: Fig. S1C-D).

Given the high number of unassigned cells under default parameters, we checked if there was differential antibody adhesion based on cell type, which if present could bias downstream deconvolution. We assigned a cell type label to all pooled cells using the CellTypist package [35] combined with unsupervised clustering (Methods). We found across tested probability thresholds that epithelial cells and fibroblasts were proportionally more likely to be unassigned in batch A, whereas T cells were proportionally less likely to be unassigned (Additional file 2: Fig. S2A). We did not observe a similar bias in batch B (Additional file 2: Fig. S2B). One difference between these two batches is that most of the unassigned cells in batch A were assigned to a single sample (id 2283) when the probability threshold was relaxed. In contrast, the newly assigned cells at lower thresholds were more evenly distributed in batch B, suggesting lower overall antibody adhesion in the cells from sample 2283. We posit that in samples where overall antibody adhesion is low, perhaps due to insufficient reagent or insufficient time for adhesion, antibodies are preferentially likely to bind to the cell surface markers of certain cell types, perhaps through greater prevalence or steric availability of CD298 and/or $\beta$2 microglobulin. Given sufficient time or reagent, however, we posit that the antibodies will eventually bind to all cell types, explaining the lack of cell type bias in other samples/batches. This emphasizes the importance of titrating reagents based on the amount of cellular input, as recommended in the cell multiplexing procol we used [36]. These results also highlight the need to scrutinize the data post-sequencing and test a range of assignment thresholds, rather than simply relying on default parameters, in order to maximize the number of confidently assigned cells. Fortunately, our data showed reasonable representation of all cell types across all posterior thresholds. This suggests that differential antibody adhesion is unlikely to have a significant impact on reference profile quality or downstream deconvolution results, except in extreme cases where no cells of a given type are labeled.

#### Genetic-based approaches lead to higher cell demultiplexing rates in HGSOC samples

We also performed genetic demultiplexing of the pooled cells. Instead of identifying sample of origin based on an experimentally added antibody, genetic demultiplexing leverages unrelated patients’ innate genetic variation to group cells based on their genotype [37]. Genotypes can be called using common variants from publicly available data, e.g., from the 1000 Genomes Project, or with genotypes called from another data modality in the same samples. The latter allows cells to be directly mapped back to samples rather than arbitrarily labeled. We used bcftools to genotype our bulk RNA-seq data [38], cellSNP-lite to genotype the single cells [39], and vireo to cluster the cells by allelic ratios of called genotypes to assign a sample of origin [40]. Under this framework, we assigned 6730 and 8866 of the cells respectively (91.4% and 90.3%) to one sample, with 558 and 705 (7.6% and 7.2%) called as multiplets and 70 and 243 (1.0% and 2.5%) unassigned (Fig. 2C, D).

Since genetic demultiplexing relies on the ability to call sample-specific genotypes for common variants within single cells, the inherent genomic instability of cancer cells has previously been an area of concern. Simulated experiments have indicated that genetic demultiplexing was possible in tumor samples [41], and these results offer confirmation in real experimental data. It has been shown that using genotypes from bulk data from the sample samples (when available) is preferable for cancer demultiplexing [41]. One could imagine that the selection strategy used for bulk data could affect results—for example, by unevenly sampling across transcripts. Here, we found that genotypes from paired bulk RNA-seq samples appear to be highly consistent across protocol types. We performed genotyping on our three bulk RNA-seq datasets (rRNA^-^ Chunk, rRNA^-^ Dissociated, polyA^+^ Dissociated) and performed genetic demultiplexing with each as a reference. We found that over 99% of cells had the same genetic demultiplexing assignment in each run (Additional file 2: Fig. S3A-C).

#### Hash and genetic demultiplexing produce highly concordant cell assignments

Encouragingly, we saw a high degree of overlap in assignments between hash and genetic demultiplexing. In cells assigned to a sample by both methods, 94.3% and 95.2% of cells were assigned to the same sample (Fig. 2E, F). The biggest area of discordance overall was that many cells were assigned by genetic demultiplexing and left unassigned by hash demultiplexing. This effect was somewhat lessened at the more permissive 80% cellranger multi threshold. We attribute the higher number of assigned cells with genetic multiplexing, even after the relaxed hash assignment threshold, to incomplete adhesion of the antibody tags. For this reason, we elected to use cells assigned by genetic demultiplexing as our single-cell reference profiles for our main deconvolution analyses.

### Dissociation causes disproportionate loss of adipocyte and other cell type markers

As mentioned previously, some cell types are more resilient to dissociation than others, which can create a bias in deconvolution when using single cells as reference profiles and in comparison of bulk to single-cell data. We assessed the effect of dissociation on tumor transcriptomic data by comparing two of our bulk RNA-seq datasets: rRNA^-^ Chunk and rRNA^-^ Dissociated.

**Fig. 3:**
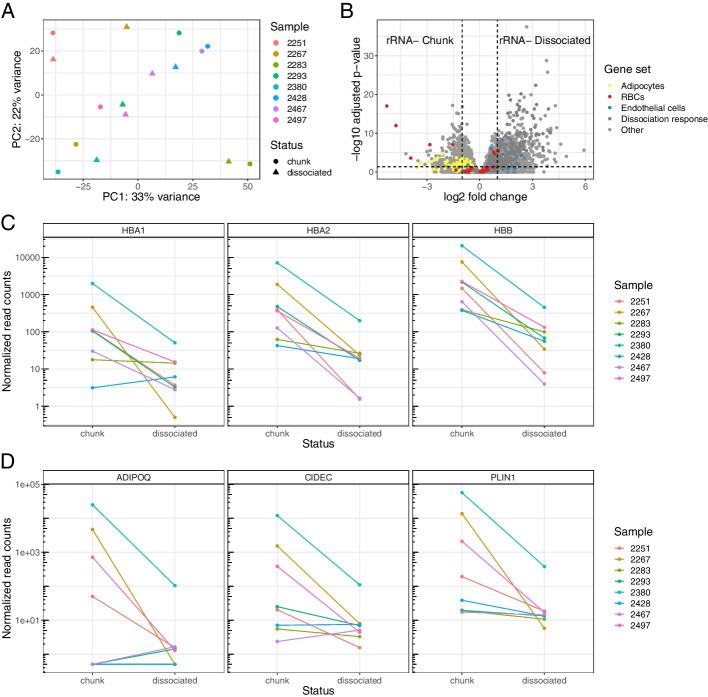
Dissociation causes disproportionate loss of red blood cells and adipocytes. **A** A principal component analysis of the rRNA^-^ Chunk and rRNA^-^ Dissociated bulk samples, where color indicates patient of origin and shape indicates dissociation status. Two points that are closer together on the PCA plot are more similar in their expression profiles. **B** A volcano plot of the differential expression results based on dissociation status, with gene sets of interest colored: genes known to be upregulated in adipocytes [45], endothelial cells [45], and red blood cells [44]. **C** Expression of hemoglobin genes in each sample based on dissociation status. **D** Expression of selected adipocyte-related genes based on dissociation status

Principal component analysis (PCA) of the samples’ expression profiles revealed that samples tended to segregate together by patient of origin in the first two principal components rather than based on dissociation status (Fig. 3A). This indicated that inter-patient heterogeneity is strongly present before and after dissociation. We ran differential expression using the DESeq2 package [42] (Fig. 3B). The genes with the highest log fold-change of expression in the tumor chunks compared to the dissociated cells were hemoglobin genes (HBA1, HBA2, HBB) (Additional file 1: Table S3). Hemoglobin genes were significantly reduced across all rRNA^-^ Dissociated samples when compared to their rRNA^-^ Chunk counterparts (Fig. 3C). Erythrocytes (red blood cells) are the predominant expressors of hemoglobin, and are lysed and removed by many dissociation protocols [43], including the one that we used. We plotted other erythrocyte-specific genes [44] and found several were significantly more abundant in the tumor chunks as well (Fig. 3B).

Several other highly increased genes in the tumor chunks were associated with adipose tissue (Fig. 3B, Additional file 1: Table S3). Adipocytes are fragile and rarely survive dissociation [46]. In a comparison of single-cell and single-nucleus RNA-seq data, adipocytes were abundant in single-nucleus data and essentially absent from single-cell data [45]. Some of our bulk RNA-seq samples expressed adipose-related genes in the tumor chunks but less in the dissociated cells. In other samples, adipose gene expression was low in both tumor chunks and dissociated cells (Fig. 3D). These data support a model where some tumors have high numbers of adipocytes, which are lost during dissociation, and others lack substantial adipose tissue. While the surgical excision site was not recorded for these samples, our data are consistent with certain samples being derived from the omentum (a layer of fat lining the peritoneal cavity to which ovarian cancer cells preferentially migrate and colonize [47]) and others from other sites.

Many of the genes that were more abundant in the dissociated cells compared to the tumor chunks are stress response pathway genes (Fig. 3B, Additional file 1: Table S4). These “signatures of dissociation” as annotated by O’Flanagan et al are highly conserved across cell and tissue types [48]; because of this, we hypothesize that stress response genes are unlikely to be selected as informative markers by a deconvolution method and the chance of introducing cell type-specific bias is low. We thus focused on how dissociation may alter cell type abundance. Gene set expression analysis using cell type signature genes from the Molecular Signatures Database [49] showed that endothelial cells, fibroblasts, macrophages, and other immune cell types are more abundant in dissociated cells (Additional file 2: Fig. S4). We confirmed increased endothelial expression using marker genes from Emont et al [45] (Fig. 3B). Indeed, many of the stromal and immune cell types one would expect to see in an HGSOC tumor are more abundant in the dissociated cells. We hypothesize this is due to increased relative abundance rather than a true biological enrichment. When red blood cells and adipocytes are disproportionately removed, the relative abundance of markers of the remaining cell types necessarily increases.

While some cell type bias in dissociated cells is caused by easily avoided technical artifacts—one could alter their dissociation protocol to not include a red blood cell lysis step—others are not easily remedied, such as the disproportionate loss of adipocytes and other fragile cell types. While adipose marker genes were not completely missing in our dissociated bulk samples, we did not find any adipocytes in our annotated paired single-cell data, consistent with the findings of [45]. Deconvolution using this single-cell data as a reference profile would, at best, be unable to detect the presence of or quantify adipocytes. This poses a particular problem for ovarian cancer studies, where adipocytes are posited to have a direct role on tumor growth and metastasis [50, 51] and explain some aspects of inter-patient heterogeneity. Other cancer types may also have other relevant cell types that are disproportionately compromised by dissociation, such as mesothelial cells [45]. Methods that assume all cell types have a reference present may exhibit unstable performance as they minimize residuals that arise from the absent cell types.

### mRNA enrichment method affects gene abundance

Many deconvolution experiments use bulk data that has been ribosomal RNA-depleted and single-cell reference profiles that have been poly-A captured. While both poly-A capture and rRNA depletion have been shown to effectively enrich for mRNA across a variety of contexts [23, 52], it is not known if this experimental difference has a downstream effect on deconvolution. Comparing two of our datasets, rRNA^-^ Dissociated and polyA^+^ Dissociated, allows us to observe the impact different mRNA enrichment methods have on gene expression profiling.

**Fig. 4:**
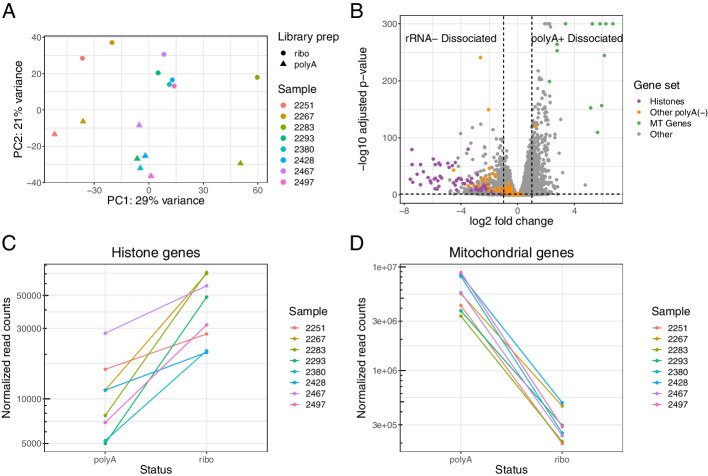
Method of mRNA enrichment affects gene expression profile. **A** A principal component analysis of the rRNA^-^ Dissociated and polyA^+^ Dissociated bulk samples, where color indicates patient of origin and shape indicates whether samples were poly-A captured or rRNA depleted. Two points that are close together on the PCA plot are similar in their expression profiles. **B** A volcano plot of the differential expression results based on mRNA enrichment method, with gene sets of interest colored: histones, other non-polyadenylated genes [53], and mitochondrial genes. **C** Combined expression of all histone genes in each sample based on mRNA enrichment method. **D** Combined expression of all mtDNA genes based on mRNA enrichment method

To visualize the differences across samples and across data types, we used PCA on a regularized log-transformed dataset comprising all genes in the rRNA^-^ Dissociated and polyA^+^ Dissociated samples. We found that the first principal component segregated samples by patient, while second principal component completely separated the rRNA^-^ Dissociated and polyA^+^ Dissociated samples from each other (Fig. 4A). The choice of mRNA enrichment method exerts a substantial effect on overall gene expression.

We performed differential expression analysis to identify trends in global expression profiles (Fig. 4B). All of top 20 most differentially abundant protein-coding genes (based on log fold change) in the ribo-depleted samples encoded histone proteins. To see if this effect was widespread among all histone genes, we aggregated their counts and found 1.7-fold to 10-fold enrichment of histone genes in the rRNA^-^ Dissociated samples compared to the polyA^+^ Dissociated samples from the same tumor (Fig. 4C). There is a simple explanation for this: canonical histone RNAs are not polyadenylated and thus missed by poly-A capture protocols [54] (the histone reads we observe in the polyA^+^ Dissociated samples are likely attributable to variant histones that are not cell cycle dependent and are polyadenylated). Several other non-polyadenylated transcripts, such as TERC (the RNA component of telomerase) and RMRP (an endoribonuclease implicated in cancer progression [55, 56]), were also highly differentially abundant in the rRNA^-^ Dissociated samples [53]. While these genes are not documented marker genes for cell types, researchers should expect that these genes will be substantially undercounted or missing in poly-A captured samples, which includes many existing tumor maps.

We observed another trend in the opposite direction: of the top 20 most differentially abundant protein-coding genes in the poly-A captured samples, 12 originated from the mitochondrial transcriptome (mtRNA) (Additional file 1: Table S6). Examining the aggregated counts of all mitochondrial RNA in both sets of samples, we found a 10-fold to 30-fold increase in mtRNA reads in poly-A samples compared to their ribo-depleted counterparts (Fig. 4D). We initially found these results surprising since most mitochondrial RNAs have no connection to ribosomal machinery. However, the widely used kit that we used for rRNA depletion has an off-target effect where non-ribosomal mitochondrial transcripts are depleted along with the mitochondrial ribosomes [57]. The apparent increased abundance of mitochondrial genes in poly-A samples is likely attributable to this technical artifact.

The percent of mtRNA reads is a major metric used for quality control of scRNA-seq data. Dissociation can result in a rupture of the cell membrane and loss of cytoplasmic RNA, causing an increase in the proportion of mitochondrial RNA [58]. Cells above a certain mitochondrial threshold are usually removed from analysis, assumed to be dead or irreparably compromised. If a researcher uses paired bulk RNA-seq that has been ribo-depleted as a reference for the expected fraction of mtRNA reads, they may choose an overly conservative threshold and lose many potentially informative cells.

### Assessing deconvolution accuracy and robustness together improves method evaluation

With more information on the effect different experimental decisions have on the data directly, we assessed the extent to which those experimental factors affect tumor deconvolution. We applied several commonly used deconvolution methods to our tumor data (Table 2) [11–15, 59]. We chose methods that return proportions of cell types, allowing us to directly compare results across methods. Each method has its own particular required inputs. In the case of methods that do not use scRNA-seq data, we used the marker gene matrices provided by the respective methods [14]. Most methods we examined require single cells as cell type reference profiles [11–13, 15, 59]. For these methods, we used cells from our pooled single-cell data that could be assigned to a sample by genetic demultiplexing and confidently annotated to a cell type (*n *= 14,608). Having two types of single-cell data from the same samples (scRNA-seq Pooled and scRNA-seq Individual) allowed us to provide the pooled cells as a reference profile and leave the individually sequenced cells to be used for validation without the pitfalls of using the same data for reference profiles and assessment.

**Table 2:** Deconvolution methods. All methods used are open source and return proportional estimates of the total composition of a tissue sample

Method	Implemented by	Uses scRNA-seq data	Availability
BayesPrism	Chu et al. 2022 [11]	Yes	R package
Bisque	Jew et al. 2020 [12]	Yes	R package
CIBERSORTx	Newman et al. 2019 [13]	Yes	Web app
EPIC	Racle et al. 2017 [14]	No	R package
MuSiC	Wang et al. 2019 [15]	Yes	R package
NNLS	Mullen and van Stokkum 2012 [59]	Yes	R package

#### Deconvolution methods have cell type bias in real and pseudo-bulk data

**Fig. 5:**
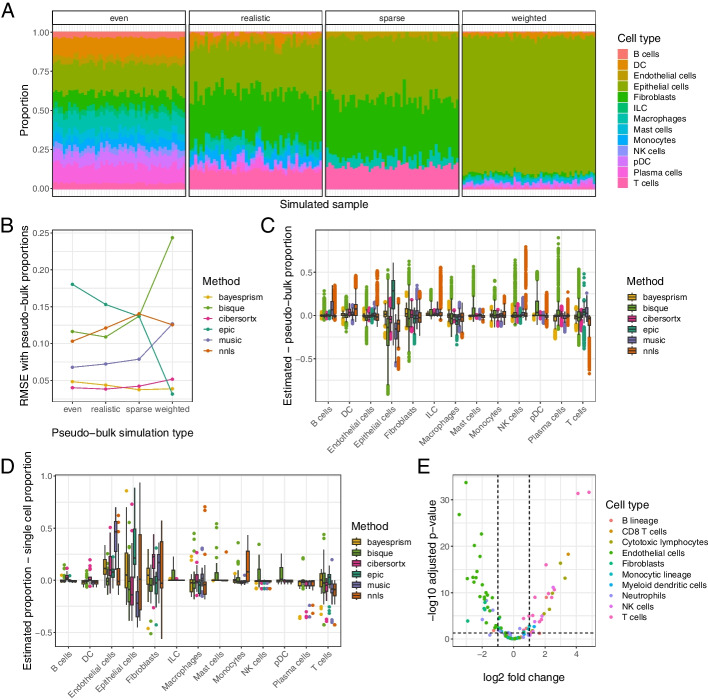
Deconvolution methods show cell type bias in real data. **A** RNA abundances from the four different simulation scenarios to generate pseudo-bulk data from our single-cell samples, shown with a single sample. For each pseudo-bulk dataset, we pre-set proportions of cells based on the simulation scenario and then randomly sampled from each cell type at those proportions. **B** The average root mean squared error (RMSE) between cell type proportions estimated by various deconvolution methods and the true simulated proportions, stratified by simulation type. **C** The difference between deconvolution estimates for our pseudo-bulk data and their true RNA abundance proportions across all simulation scenarios, stratified by cell type. A score of 0 indicates perfect concordance between the deconvolution estimate and the true pseudo-bulk value. **D** The difference between deconvolution estimates for our real data and the proportion of RNA reads mapping to that cell type in the corresponding sample’s single-cell data. **E** A volcano plot of differential expression in cell type markers between pseudo-bulk (non-simulated, aggregating all reads from all single-cells in the sample) and polyA^+^ Dissociated real bulk data. Genes are selected and colored based on the signature matrix from MCPcounter [61]

We generated pseudo-bulk samples using 7 of our scRNA-seq Individual samples, using cells annotated by cell type. We excluded sample 2428 due to an insufficient number of cells. We used SimBu [60] to create four datasets of 50 pseudo-bulk samples each, spanning a range of potential scenarios that a deconvolution method should be able to accurately characterize (Fig. 5A). Each pseudo-bulk sample consisted of count data from 2000 single cells, sampled according to the scenario parameters. One scenario mirrored the proportions of cell types observed in the single-cell samples; we will refer to this scenario as “realistic.” Another scenario had approximately equal numbers of all the cell types present in the single-cell data; we will call this scenario “even.” A scenario we will call “sparse” only included cell types believed to be common in our tumor dataset (epithelial cells, endothelial cells, fibroblasts, macrophages, and T cells), to enable us to assess how deconvolution methods handle absent cell types. One of the scenarios, called “weighted,” is designed to mimic our expectation that many epithelial tumors are predominated by cancer cells; in this scenario, the epithelial cell fraction was held constant at 70% with random proportions of other cell types. Since cell types have different amounts of mRNA contents, most deconvolution methods predict proportions of each cell type’s mRNA contribution, not proportions of total cells. We therefore used proportion of transcriptional reads deriving from sampled cells of each cell type as a ground truth for our pseudo-bulk accuracy analysis (Fig. 5A).

We ran the deconvolution methods on all of the simulated pseudo-bulk datasets and calculated the room mean squared error (RMSE) between the estimated and known pseudo-bulk proportions. We found that performance differed both across methods and across simulation types, with BayesPrism and CIBERSORTx having the lowest and most consistent errors overall (Fig. 5B). We next investigated whether some methods gave better estimates of certain cell types than others in pseudo-bulk data. We stratified the proportion estimates by method and cell type and subtracted the corresponding pseudo-bulk mRNA proportion values from them (Fig. 5C). For almost all cell types, all methods had a mean difference between true and estimated proportions of approximately 0, albeit with frequent outliers. The one exception was epithelial cells, whose mRNA abundance was overestimated by three of the six methods.

Given that we had paired bulk and single-cell data, we could compare our pseudo-bulk results to the output of deconvolution on real bulk data for each sample. We ran deconvolution on each of our three bulk data types (rRNA^-^ Chunk, rRNA^-^ Dissociated, and polyA^+^ Dissociated). We used the number of cells of each type from the individual single-cell data to approximate proportions, with the assumption that deconvolution results that are closer to the proportions from the single-cell data will be closer to the unknown true proportions comprising the bulk data (we excluded sample 2428 from this analysis because of its small number of captured single cells). We subtracted proportions estimated from our single-cell data from the deconvolution-estimated proportions for our bulk data (Fig. 5D). In this comparison, we found that most methods undercount macrophages and T cells and most overcount endothelial cells, epithelial cells, and fibroblasts. The difference between most other cell types’ estimated bulk and matched single-cell proportions was close to 0, which is expected given that these cell types are comparatively rarer and so variance is naturally smaller. These trends occur across each of the bulk data types (Additional file 2: Fig. S5A-C).

The concordance across methods in undercounting certain cell types and overcounting others in the real bulk data led us to speculate that it may derive from a true difference in cell type proportions between bulk and single-cell data. To explore this, we ran differential expression analysis on our polyA^+^ Dissociated bulk data compared to our pseudo-bulk data generated from all scRNA-seq Individual cells. Using polyA^+^ Dissociated bulk data to compare to pseudo-bulk ensured that any differences in gene expression were not an artifact of dissociation status or method of mRNA enrichment. We found a high number of differentially expressed genes, suggesting that discrepancies between bulk and single-cell data extend beyond the experimental design decisions we controlled for. We filtered differential expression results to the cell type unique markers used by MCPcounter [61]. T cell markers were all more expressed in the single-cell data than in the bulk, and the overwhelming majority of fibroblast and endothelial cell markers were more expressed in the bulk data than in the single-cell (Fig. 5E). These results suggest that some step in the technical protocol post-dissociation also creates a cell type-specific bias in what cells are captured by scRNA-seq. We used microfluidic-based scRNA-seq, so loading the cells into microfluidic droplets could be differentially efficient. Endothelial cells and fibroblasts are irregularly shaped and highly integrated into the extracellular matrix (ECM) and vasculature; these groups of cells may be more prone to incomplete dissociation and being strained prior to loading. This may be particularly true in the context of a high-grade tumor, where cancer cells establish a dense and highly disorganized ECM and vasculature compared to normal tissue. In contrast, T cells are more spherical and inherently migratory and are thus more likely to be dissociated and loaded efficiently. Additionally, it has been reported that some kinds of T lymphocytes are underrepresented in deconvolution methods that use marker genes [62]. Perhaps the phenotypic heterogeneity observed in most T cell lineages makes it harder to identify a unifying expression profile for accurate quantification.

Regardless of the cause of the cell type bias in scRNA-seq, its presence suggests an uncomfortable truth: bulk and single-cell RNA-seq are substantially different modalities. This challenges the use of accuracy on pseudo-bulk data as a gold standard for deconvolution because performing well on pseudo-bulked single-cell data does not necessarily equate to performing well on real bulk data. It also suggests that comprehensive profiles of the tumor microenvironment should include both bulk and single-cell assays to allow accurate analysis of the TME.

#### Deconvolution methods vary in robustness to technical differences

We propose an additional way to evaluate deconvolution methods: robustness of results to different experimental and protocol decisions. We posit that a method that returns consistent results for the same tissue sample, regardless of what kind of pre-sequencing processing is done and what reference profile it is given, is likely to give meaningful results across a range of real-world settings and studies. The concept of robustness has been previously employed in deconvolution with the assertion that constructing better marker gene matrices requires taking cross-microarray platform variation into account [63]. Here, we extend this concept to single-cell informed deconvolution methods.

**Fig. 6:**
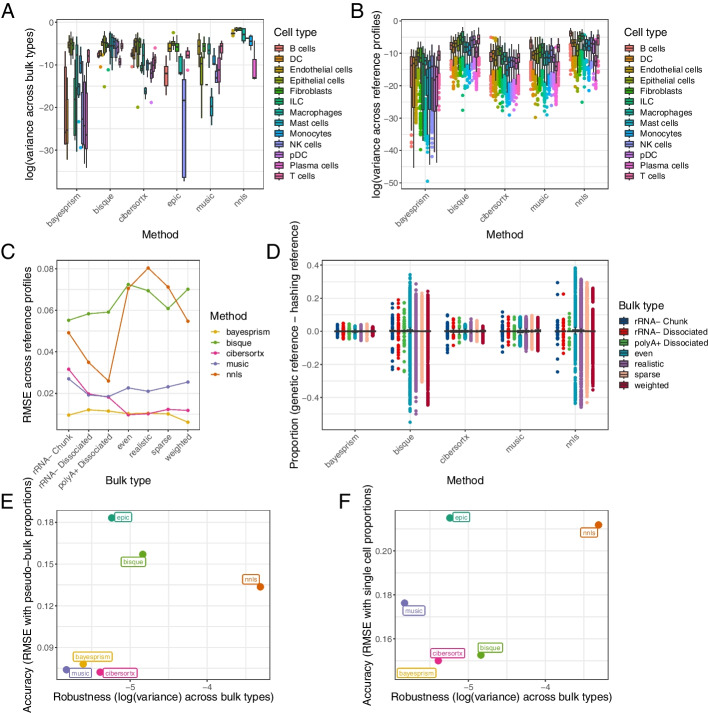
Deconvolution methods vary in robustness to changes in bulk and single-cell data. **A** Variance of deconvolution results across bulk data type. For each method, we calculated the variance between the estimated proportion for a given cell type in a given sample in the rRNA^-^ Chunk, rRNA^-^ Dissociated, and polyA^+^ Dissociated data. **B** Variance of deconvolution results across reference profile size. For each method, we calculated the variance between the estimated proportion of a given cell type in a given sample when using cells assigned by genetic demultiplexing (*n *= 14,608) as a reference vs. using cells assigned by antibody-based demultiplexing with default parameters (*n *= 7574). **C** The average RMSE between cell type proportions using a smaller and larger reference profile. **D** The average difference between cell type proportion estimates using the smaller vs. the larger reference profile, stratified by bulk/pseudo-bulk data type. **E** The final accuracy vs. robustness result for each method based on pseudo-bulk data, with variance in estimates for bulk data types and RMSE between estimate and simulated proportions for pseudo-bulk data. **F** Accuracy vs. robustness of each method based on true bulk data, with variance in estimates for bulk data types and RMSE between real bulk estimate and real single-cell proportion

Since our three bulk-sequenced datasets originated from the same tumors, we would expect a robust deconvolution method to return similar cell type proportions for a given tumor using each bulk dataset as input. We compared the variance in proportion estimates for each combination of sample, cell type, and method (e.g., the proportion of B cells CIBERSORTx reported for sample 2251 in rRNA^-^ Chunk vs. rRNA^-^ Dissociated vs. polyA^+^ Dissociated data) (Fig. 6A). The more abundant cell types in our tumors, such as endothelial cells and epithelial cells, had naturally higher variance than the less abundant cell types, such as NK cells and plasma cells. MuSiC had the lowest variance overall, followed by BayesPrism.

As we have already demonstrated, changes in how the single-cell data are generated can change the cell type representation of the reference profile, which can skew deconvolution results. We used the results from our demultiplexing experiment to determine what deconvolution methods are more robust to technically driven changes in the single-cell reference profile. We ran deconvolution on our bulk data using a reference comprising only the cells assigned by hash demultiplexing at the default 90% probability threshold. This represented 51.8% of the cells used in our original profile of cells assigned by genetic demultiplexing. Given that each cell type was still reasonably represented in the smaller single-cell dataset, we would expect a robust method to return similar deconvolution results using either reference profile (note that these analyses only apply to deconvolution methods that use single-cell reference profiles, so methods that use pre-selected marker genes were excluded).

We compared the variance across the single-cell profiles in each combination of sample, cell type, method, and bulk type (e.g., the proportion of B cells CIBERSORTx reported for sample 2251’s rRNA^-^ Chunk data using the genetic demultiplexed reference profile vs. the hash demultiplexed reference profile) (Fig. 6B). We found BayesPrism had lower average variance across most cell types, with CIBERSORTx having the next lowest, indicating these methods may be more invariant to slight changes in the reference profile. We next calculated the RMSE between the deconvolution results across the two reference profiles (Fig. 6C). BayesPrism and CIBERSORTx had very low error across all pseudo-bulk types, but BayesPrism’s errors were lower on the true bulk data. MuSiC performed similarly to CIBERSORTx on true bulk data but had higher errors on pseudo-bulk data, with Bisque and NNLS having the highest and most variable errors. Seen another way, the difference between the deconvolved cell type proportions using either reference profile was relatively small and uniform across bulk data types for BayesPrism, slightly larger but still uniform across bulk types for CIBERSORTX and MuSiC, but highly variable in Bisque and NNLS, particularly in the pseudo-bulk types (Fig. 6D).

Halving the size of the reference profile by using the default assignments from Cell Ranger multi offered some evidence of how changes in reference profile size affects deconvolution results, but even then, the reference profile had thousands of cells with even rare cell types represented in the dozens or more. To assess how deconvolution methods handle even smaller reference profiles, we proportionately downsampled our pooled single-cell data into several simulated reference profiles, having as few as 200 total cells (Methods). We ran each of the deconvolution methods on our bulk and pseudo-bulk data using these new simulated reference profiles. We found that some methods have built-in conditions on the size of the reference profile for rare cell types: MuSiC will only quantify a cell type with 2 or more cells in the reference profile, whereas CIBERSORTx will only quantify those with 3 or more cells. Finally, we calculated the variance of deconvolution proportion estimates in a stepwise fashion, including results from a smaller reference profile each time. (Additional file 2: Fig. S6) While all deconvolution methods produced less consistent results when smaller reference profiles were used, evidenced by a higher average variance, BayesPrism still had lower variance than the other methods when all sizes of reference profile were considered, with CIBERSORTx a close second. We determined that these methods are likely to be more robust to variations in the reference expression profile driven that are driven by a small sample size.

Finally, to consider robustness and accuracy simultaneously, we plotted a metric of each on an axis of a graph (Fig. 6E, F) to determine if there was a tradeoff between methods, i.e., if some methods return precise-but-not-accurate results across experimental conditions and some methods are accurate under some experimental conditions but not robust. We used variance across true bulk types as the robustness axis, and for the accuracy axis, we used either the RMSE between estimates and pseudo-bulk proportions (Fig. 6E) or RMSE between real bulk proportions and single-cell proportions (Fig. 6F). BayesPrism and CIBERSORTx scored well on both axes, and while MuSiC had a slightly better robustness score and had good accuracy (low RMSE) on pseudo-bulk data, it had poor accuracy (high RMSE) on true bulk data.

### Relative impact of experimental factors on deconvolution results

In this paper, we have considered several different experimental factors that can alter the results of a deconvolution analysis: whether or not the bulk tissue is dissociated prior to sequencing, whether rRNA depletion or poly-A capture is performed, the size of the single-cell dataset used as a reference profile, and the choice of deconvolution method used. In order to contextualize the ultimate impact each of these experimental factors has on deconvolution, we performed an analysis of variance (ANOVA) using each of the experimental factors mentioned as a potential explanatory variable for deconvolution results. Since proportional estimates of each cell type must sum to 1 and are therefore not independent, we limited our analysis to the proportions of the single most variable cell type, which was epithelial cells. Unsurprisingly given the variation seen in cell type proportions across methods, choice of deconvolution method was the factor with the most significant effect on epithelial cell proportion estimates (*F* = 53.406, *p* < 2e−16). The choice of an mRNA enrichment method (rRNA depletion or poly-A capture) also had a significant effect on epithelial cell proportion estimates (*F* = 56.628, *p* = 1.55e−13). The size of the single-cell reference profile used was not significant (*F* = 1.056, *p* = 0.383), suggesting that—barring extremely small reference profiles where entire cell types are missing—deconvolution methods are largely invariant to small changes in the single-cell reference profile. Dissociation status of the bulk data was also not significant (*F* = 0.316, *p* = 0.574). This implies that dissociation does not affect deconvolution methods’ ability to identify and quantify cell type signals, with the important caveat of cell types like adipocytes that are compromised enough by dissociation to be entirely missing from the reference profile.

This significant effect of mRNA enrichment method on epithelial cell estimates was initially surprising, given that we did not find cell type-specific differences driven by mRNA enrichment. However, when we compared the bulk profiles of the rRNA^-^ Dissociated and polyA^+^ Dissociated samples (Fig. 4), samples segregated efficiently by mRNA enrichment method in a principle component analysis. Also, there were more significantly differentially expressed genes between rRNA^-^ Dissociated and polyA^+^ Dissociated samples (7588 genes with log fold-change (LFC) > 0 and 7384 LFC < 0, FDR = 0.05) than between rRNA^-^ Chunk and rRNA^-^ Dissociated (3142 LFC > 0 and 2694 LFC < 0, FDR = 0.05). From this, we intuit that large global changes in gene abundance are sufficient to bias a deconvolution method, even without cell type-specific changes.

## Discussion

In this study, we designed a unique experiment profiling HGSOC tumors in multiple ways to allow for direct characterizations of how experimental design affects the deconvolution of cancer data. We introduce the metric of robustness across experimental protocols to deconvolution methods to ensure results are consistent for a single tumor independent of the technical choices made. Performing these analyses on real tumor data instead of simulated data establishes a model dataset with which future deconvolution methods can be evaluated for robustness.

We applied and evaluated six different deconvolution methods for both accuracy and robustness. We intend this to be an examination of how different commonly used existing methods can vary in robustness and not a comprehensive benchmark. We invite researchers to use this dataset to evaluate the robustness of other existing and future methods. We have included a tutorial on GitHub for running new methods on this data (Availability of data and materials).

Our analysis focused on deconvolution methods that return absolute proportions of cell types within a sample. Other common methods return unitless scores that can be compared across samples to assess relative abundance but which do not indicate an absolute proportion of cell types in the sample. We initially applied several such methods to our data (Additional file 1: Table S7) [61, 64–69]. However, when we attempted to assess the accuracy of these methods on our bulk data, based on their correlation with the proportions in the single-cell data, correlation values were very low (Additional file 2: Fig. S7A-G). Many of these methods focus on granular profiling of the immune compartment rather than total deconvolution, so our dataset may not be optimal for evaluating such methods.

Methods development for deconvolution is an active area of research. As such, we offer recommendations for researchers designing the next generation of deconvolution methods. One major consideration brought to light by this study is that certain cell types are present in the bulk tissue but lost from single-cell data. These cell types are thus unquantifiable by existing reference profile-based deconvolution methods. At a minimum, we recommend that future methods include a parameter to capture the proportion of “unknown cells” that lack a reference within a sample to quantify missing cell types indirectly (this is already implemented in certain methods, such as EPIC [14]). Alternatively, a potential area for development would be a method that employs single-nucleus data (snRNA-seq) as a reference profile for deconvolution. Some cell types that are lost by dissociation can still be profiled using snRNA-seq due to the protection of the nuclear membrane [45]. A method that corrects for differences between nuclear and cytoplasmic RNA may effectively leverage an snRNA-seq reference profile to more accurately characterize all cell types present in a given tissue. Another option would be a combination of reference profile and marker gene strategies, using reference profiles for cell types that can be single-cell sequenced and cell type markers obtained from the literature or from bulk sequencing for cell types lost in single-cell sequencing. While it does not seem like an arbitrarily large reference profile is necessary for accurate deconvolution, combining single-cell data across tissue contexts and platforms from public datasets such as the Human Cell Atlas may also allow for better quantification of rare cell types [70]. How to account for the differences in cell type gene expression across different tissue contexts is a promising area for future research.

Regardless of individual algorithmic decisions, developers of new deconvolution methods should be sure to test on real bulk and single-cell datasets that have been prepared using representative experimental protocols. As we have shown here, different design decisions each introduce biases that can affect deconvolution. Testing on only one data type renders these biases invisible. Our results show that pseudo-bulk data is an inherently limited metric and should not be used as the solitary gold standard for evaluation. Also, a recent study by Hu and Chikina confirms that the traditional way of simulating data for evaluating deconvolution does not adequately represent biological heterogeneity and proposes new ways for better capture heterogeneity in simulation [71]. By incorporating robustness evaluations across both well-designed simulations and real datasets into their testing process, researchers can maximize the utility of their method across many future research questions.

We also have recommendations for scientists interested in designing an experiment to use deconvolution to profile the TME. We note that our study focused on a single tumor type, HGSOC, so results may vary for other tumor types, but we believe that these principles are likely to be helpful for many kinds of heterogeneous solid tumors. For those generating novel data, pooling is an effective way to single-cell profile more tumors at a considerably reduced cost. We recommend using genetic demultiplexing to assign cells back to their sample of origin since it is independent of the efficiency of antibody loading and thus results in fewer unassigned cells with no observed bias by cell type. Where genetic demultiplexing is not possible, e.g., using multiple samples from the same patient or genetically related patients, a study by Howitt et al. offers recommendations of alternate software packages for hash demultiplexing, even in scenarios with low quality hashtag data [72].

Method of mRNA enrichment appears to be a key consideration when designing a bulk sequencing protocol for tumor deconvolution. For those generating novel bulk sequencing data, we recommend performing poly-A capture to align more closely with the single-cell reference profile. That said, choosing a robust deconvolution method should allow for high performance when using either poly-A captured or rRNA-depleted samples. Out of all of the methods we tested, BayesPrism had the highest combination of robustness (across bulk expression protocols and single-cell reference profile sizes) and accuracy (compared to true pseudo-bulk proportions and real single-cell data).

## Conclusions

Our results indicate that differences in data generation protocols introduce biases that alter the output of most deconvolution methods. This is true across protocols within a single data modality, such as bulk RNA sequencing of dissociated vs. non-dissociated tissue, but it is also true across different data modalities, namely bulk vs. single-cell RNA sequencing. Even when mRNA enrichment methods and dissociation status are the same, bulk and pseudo-bulk single-cell data have cell type specific abundance differences. From this, we intuit that characterizing the true cell type profile of a tissue is more complex than is deconvolving a collection of single cells that have been pseudo-bulked. Thus, accuracy on pseudo-bulk data is more of a silver standard than a gold standard. A well-performing deconvolution method will need to balance the trade-off between accuracy and robustness, being careful not to overfit to either silver standard. Out of the methods we tested, BayesPrism had the highest combination of robustness and accuracy. Development of even more robust deconvolution methods, as well as thoughtful design of experiments to generate data for deconvolution, will allow for high-quality characterizations of the TME across hundreds or thousands of samples in bulk datasets. These large sample sizes will enable a better understanding of the fundamentals of tumor biology at a population level and potentially identify opportunities for novel targeted therapeutics.

## Methods

### Experimental methods

#### Tumor processing/dissociation

Samples were collected from 8 patients with HGSOC by the University of Pennsylvania Ovarian Cancer Research Center’s Tumor BioTrust Collection (RRID: SCR_022387). All patients underwent primary debulking surgery and had not received neoadjuvant chemotherapy. A 10X enzymatic digest stock solution was made by combining a 500 mL bottle of RPMI-1640 (Gibco 61870036), 1000 mg collagenase (Millipore Sigma C9407), and 150 KU DNase type IV (Millipore Sigma D5025). Solution was sterile filtered, aliquoted, and stored at − 20° C until use. Tumor samples were minced into 1mm pieces. Portions of the tumor were flash frozen. Remaining fresh tissue was put into a 1X solution of the enzymatic digest solution, diluted with RPMI-1640. Tumor tissues were dissociated overnight at room temperature. Dissociate mixture was filtered using a sterile 100 $\mu$m mesh filter and washed using DPBS. Red blood cells were removed using ACK Lysis Buffer. Dissociated cells were resuspended in 90% human AB serum/10% DMSO freezing media and frozen at − 80° C in a freezing chamber then transferred to − 150° C for long term storage.

#### Multiplexing

We grouped samples into two batches of four samples each, in order to balance the cost-saving potential of multiplexing with minimizing the risk of clogging by loading cells from all eight samples at once. For each set of four samples, a portion of the dissociated cells was thawed and labeled with TotalSeq-B anti-human antibody-oligonucleotide conjugates from BioLegend, which are designed to label most cells via binding to both CD298 and $\beta$2 microglobulin. The cells were then pooled and prepared for sequencing using the 10X Genomics 3’ CellPlex Kit.

#### Single-cell sequencing

We performed scRNA-seq using the Chromium Next GEM platform from 10X Genomics. We loaded our thawed dissociated cells into emulsified droplets with Single Cell 3’ v3.1 Gel Beads using the Chromium Next GEM Chip G. We then added primers complete with a unique molecular identifier (UMI) and a poly-dT sequence to ligate with the mRNA molecules in each droplet and generate cDNA. Droplets were then broken, pooling the labeled cDNA for amplification, fragment size selection, and sequencing.

The multiplexed single-cell samples were prepared in the same way as above, but additional primers mapping to the cell surface protein feature barcodes were added alongside the other primers, allowing for specific amplification of the antibody-associated oligonucleotides.

All single-cell samples, multiplexed and individually run, were sequenced on an Illumina NovaSeq 6000 system using the S2 Reagent Kit v1.5 (100 cycles).

#### Bulk sequencing

For each sample, we bulk sequenced thawed tumor chunks; we also bulk sequenced a portion of the thawed dissociated cells in two ways: (1) tumor chunks and one set of dissociated cells were prepared following Illumina’s TruSeq Stranded Total RNA protocol. Ribosomal RNA was depleted using the Illumina Stranded RiboZero Plus kit. Then, cDNA was synthesized from the remaining RNA and enriched using PCR. (2) Another set of thawed dissociated cells were prepared according to Illumina’s TruSeq Stranded mRNA protocol. In this protocol, mRNA molecules attach to oligo-dT magnetic beads for purification before cDNA synthesis and enrichment.

All bulk samples were sequenced on an Illumina NovaSeq 6000 system using an S2 Reagent Kit v1.5 (300 cycles).

### Computational methods

#### Data processing

The single-cell data were processed using 10x Genomics’ Cell Ranger software version 6.1.2. The raw sequence files were converted to FASTQ files using the cellranger mkfastq function, which were then aligned, filtered, counted, and converted into a gene by cell matrix by the cellranger count function. These samples were aligned using a GrCh38 reference genome provided by 10x Genomics (2020-A).

The bulk data were processed using two different aligners in order to account for some deconvolution methods requiring raw read counts and others requiring transcripts per million (TPM): (1) for methods requiring raw read counts, we processed the samples using the STAR aligner version 2.7.10 [73], using an index generated from the same reference genome as the single-cell data (10x Genomics GrCh38 2020-A). We used STAR’s quantMode parameter to generate per-gene read counts for each bulk sample. (2) Since calculating transcripts per million requires consideration of transcript length, we also quantified the bulk samples using salmon version 1.9.0 [74]. We used an index generated from the GENCODE release 32 reference transcriptome (GRCh38.p13). Salmon returns per-transcript quantifications, which we combined across transcripts of a gene to get per-gene TPM values.

#### Demultiplexing

For the pooled single-cell data, we quantified and separated the cells by sample of origin using Cell Ranger version 6.1.2, specifically the function cellranger multi. In addition to the normal alignment and cell counting steps, cellranger multi also quantifies the provided cell multiplexing oligos (CMOs) and splits each cell’s read values into one of several matrices: one for each provided CMO and one for cells that were not able to be assigned at the given threshold. It also generates a unique BAM alignment file for the reads in each matrix and an assignment report giving the probability estimates for each barcode being assigned to each particular sample, called as a multiplet, or called as a blank droplet.

For the genetic demultiplexing, we first genotyped the STAR-aligned bulk data using the mpileup and call functions of bcftools version 1.7 [38]. To genotype the single-cell data, we used the BAM files generated by cellranger multi, concatenated into a single file. We genotyped this file using cellsnp-lite version 1.2.2 [39] with the variant calls from the bulk data as a reference for sites of heterogeneous genotypes across samples. We used vireo version 0.5.7 [40] to assign cells to a donor group based on the cellsnp-lite genotypes.

#### Single-cell processing and annotation

We used miQC to identify a sample-specific threshold using percent mitochondrial reads and library complexity (number of unique genes expressed) to filter out dead and compromised cells [75]. All cell counts reported in the paper are from after this filtering step.

We assigned cell type labels to our single-cell data, both scRNA-seq Individual and scRNA-seq Pooled, using a combination of unsupervised clustering and CellTypist [35]. For each sample and pool, we ran unsupervised clustering using the scran (version 1.24.1) and igraph (version 1.3.5) packages in R [76, 77]. Per-cluster cell type annotations were defined using marker genes, via the findMarkers function in scran. We ran CellTypist version 1.1.0 with overclustering to converge similar cells to a single cell type assignment. Cells in the pooled samples with concordant assignments based on unsupervised clustering and CellTypist were used as the default reference profile for all single-cell-based deconvolution methods.

#### Bulk differential expression

Differential expression analysis was done using the DESeq2 package in R (version 1.36.0) [36]. For each analysis, we compared the eight samples from each condition (rRNA- Chunk vs. rRNA- Dissociated and rRNA- Dissociated vs. polyA+ Dissociated respectively). The sample of origin was used as a covariate to control for sample-specific effects. Principal component analysis of the bulk samples was also done using DESeq2.

#### Pseudo-bulk data generation

We used SimBu version 1.0.0 [60] as a way to efficiently sample single cells by cell type. For each scenario (even, realistic, sparse, weighted), SimBu calculated the appropriate percentage of each cell type for the custom designed scenario. For each scenario, we simulated 50 samples out of each scRNA-seq Individual sample (*n *= 7, sample 2428 excluded). Across each simulated sample, SimBu added a random noise parameter to each cell type and then recalculated the proportions to sum to 1. It then multiplied these percentages by the desired number of cells, converted it to an integer, and then randomly sampled with replacement from the labeled single cells of that cell type. The reads from all sampled cells were then combined to form a pseudo-bulk sample. SimBu also offers a correction for different cell types having different amounts of mRNA in the form of scaling factors, but we did not use these in our analysis in order to preserve the integer read counts. Instead, to account for cell type differences in mRNA abundance, we calculated the fraction of transcriptional reads contributed by each of the sampled cells, aggregated them by cell type, and used the proportion of RNA contributed as the pseudo-bulk fraction rather than the proportion of cells.

#### Deconvolution

We created a snakemake pipeline [78] to run each deconvolution method on our various real and pseudo-bulk samples. We used cells from the scRNA-seq Pooled samples as a reference profile for those methods that require it. We implemented the methods that return cell type scores using the immunedeconv R package [16].

#### Simulated reference profile generation

We generated simulated reference profiles with desired sizes of 2000, 1000, 500, and 200 cells. We calculated the proportions of each cell type in our full pooled single-cell dataset and had each simulated dataset mirror these proportions, with a minimum of one cell per cell type (note that rounding the desired numbers of cells to integers meant a slightly different number of total cells in some profiles than the named simulation size). For each cell type, we randomly sampled cells without replacement from all labeled cells of that type.

### Supplementary Information

**Additional file 1: Supplemental Tables 1–8.**

**Additional file 2: Supplemental Figures 1–7.**

**Additional file 3.** Review history.

## Data Availability

The dataset supporting the conclusions of this article is available in the Gene Expression Omnibus (GEO) (processed gene count tables) under accession GSE217517 [79] and Database of Genotypes and Phenotypes (dbGaP) (raw FASTQ files) under accession phs002262.v2.p1 [80]. The code for all the analyses performed in this paper is available at https://github.com/greenelab/deconvolution_pilot under a BSD-3-Clause license [81]. It is also available through Zenodo under DOI 10.5281/zenodo.8475 [82].
