# Supplementary material for: Performance of computational algorithms to deconvolve heterogeneous bulk ovarian tumor tissue depends on experimental factors
Source: Genome Biol. 2023 Oct 20;24:239. doi: 10.1186/s13059-023-03077-7 (PMC10588129; doi:10.1186/s13059-023-03077-7)
Supplement: Supplementary file 1 — Additional file 1: Supplemental Tables 1–8. [file 13059_2023_3077_MOESM1_ESM.pdf]

Supporting Information for

Performance of computational algorithms to deconvolve heterogeneous bulk tumor tissue depends on experimental factors

Hippen, Omran, Weber, Jung, Drapkin, Doherty, Hicks, and Greene

Casey S. Greene, e-mail: casey.s.greene@cuanschutz.edu

This PDF file includes:

Tables S1 to S8

Table S1. Number of demultiplexed cells, Batch A

Assignment	Hash (90% probability)	Hash (85% probability)	Hash (80% probability)	Genetic
2380	920	958	966	1010
2267	1119	1149	1159	1106
2283	578	1204	1737	2862
2293	1629	1663	1674	1720
Unassigned	2823	2095	1533	48
Multiplets	286	286	286	612

Table S2. Number of demultiplexed cells, Batch B

Assignment	Hash (90% probability)	Hash (85% probability)	Hash (80% probability)	Genetic
2428	0	15	178	306
2251	1215	1220	1225	3773
2467	1095	1126	1137	1904
2497	1424	1470	1492	2942
Unassigned	5935	5838	5637	150
Multiplets	145	145	145	739

Table S3. 20 most downregulated protein-coding genes in rRNA⁻ Dissociated samples compared to rRNA⁻ Chunk samples. Genes associated with erythrocytes and adipocytes are indicated.

Gene	Mean normalized counts	$\log_2$ Fold Change	BH-adjusted p-value	Notes
HBB	2264.20801	-5.274949	9.875591e-18	Erythrocytes
HBA2	673.75110	-4.757030	1.107237e-12	Erythrocytes
CXCR1	81.30936	-4.189585	2.141483e-05	
HBA1	181.66259	-3.916449	2.709565e-04	Erythrocytes
LEP	659.43123	-3.501804	1.399598e-03	
PLIN1	4600.18340	-3.360219	5.274116e-03	Adipocytes
CIDEC	886.53878	-3.343077	3.829733e-03	Adipocytes
TEX48	2.97750	-3.111579	1.172056e-02	
FCGR3B	193.80341	-2.994792	5.661478e-06	
CXCR2	123.26475	-2.902115	4.611948e-05	
GPD1	2110.00336	-2.864240	2.950040e-03	Adipocytes
ALAS2	39.75417	-2.832402	8.931344e-08	Erythrocytes
THRSP	111.35135	-2.806047	3.066181e-06	
TMC2	18.98199	-2.726750	4.035121e-04	
AL845331.2	29.66942	-2.685222	1.105572e-02	
ACVR1C	402.78381	-2.561761	6.530874e-04	Adipocytes
SLC7A10	173.51749	-2.541003	3.104534e-03	
BOD1L2	9.44344	-2.524437	4.159761e-02	
SERPINI2	37.79487	-2.516106	4.493992e-03	
BLID	10.53601	-2.486332	7.208722e-05	

Table S4. 20 most upregulated protein-coding genes in rRNA- Dissociated samples compared to rRNA- Chunk samples. Genes known to be part of dissociation response are indicated.

Gene	Mean normalized counts	$\log_2$ Fold Change	BH-adjusted p-value	Notes
PYDC1	16.081991	5.920640	2.510381e-06	
PRR35	5.705105	4.920525	9.804939e-05	
AGXT	42.060301	4.605439	6.884650e-08	
EREG	1495.336882	4.372911	3.934243e-12	
CSF3	840.783269	4.324277	2.777384e-03	
ASTL	483.145817	4.295618	8.735476e-18	
HSPB3	13.932159	4.284497	1.493207e-05	
SELE	1265.716907	4.235680	5.918690e-04	
IL1B	5013.558651	4.040173	5.868073e-12	
TRIM72	51.103458	4.034666	1.915679e-11	
CXCL8	9524.606822	4.014204	8.538524e-11	Dissociation response
CTRB2	33.088482	3.981734	6.685684e-06	
KRT17	7909.537227	3.956979	4.803680e-11	Dissociation response
RNF223	70.229533	3.920271	3.549464e-06	
PMAIP1	1407.230059	3.904967	5.615352e-17	Dissociation response
CSF2	27.287446	3.879749	4.848755e-08	
ADRA2B	329.169429	3.857407	1.808425e-26	
CCL3L1	1773.033535	3.852048	1.466380e-11	
SLC32A1	2.625897	3.847003	3.499084e-02	
DLX2	6.076595	3.829267	5.019865e-04	

Table S5. 20 most downregulated protein-coding genes in polyA$^+$ Dissociated samples compared to rRNA$^-$ Dissociated samples. Histone and other non-polyadenlyated genes are indicated.

Gene	Mean normalized counts	log_2 Fold Change	BH-adjusted p-value	Notes
HIST1H4F	126.165209	-7.584147	3.336182e-28	Histones
HIST1H1E	3875.456278	-7.466552	2.976823e-80	Histones
HIST1H3I	97.949551	-7.450876	8.066728e-54	Histones
HIST1H4L	28.497041	-7.407631	7.878764e-23	Histones
HIST1H4B	195.566155	-7.098757	8.131410e-37	Histones
HIST1H4A	130.716736	-6.862988	1.656545e-18	Histones
HIST1H3F	259.470135	-6.856573	7.531311e-54	Histones
HIST1H1B	658.088415	-6.657982	2.935287e-41	Histones
HIST1H2BI	183.101743	-6.321904	3.551740e-31	Histones
HIST1H4C	319.913332	-6.170677	3.478121e-25	Histones
HIST1H3B	446.961627	-6.117763	8.645489e-22	Histones
HIST1H2AL	99.268990	-5.958138	1.898573e-63	Histones
HIST1H2AH	167.281173	-5.886553	2.006220e-28	Histones
HIST2H3C	7.649181	-5.836300	2.089754e-11	Histones
HIST1H1D	1299.603334	-5.819167	6.793016e-54	Histones
HIST1H1A	44.148276	-5.803621	6.418551e-30	Histones
HIST2H3A	7.052000	-5.689888	1.010101e-10	Histones
HIST1H2BB	97.452607	-5.633236	2.247278e-25	Histones
HIST1H2BO	196.279002	-5.567126	1.466021e-56	Histones
HIST1H3C	145.699517	-5.561191	1.495439e-15	Histones

Table S6. 20 most upregulated protein-coding genes in polyA$^+$ Dissociated samples compared to rRNA$^-$ Dissociated samples. Mitochondrial genes are indicated.

Gene	Mean normalized counts	$\log_2$ Fold Change	BH-adjusted p-value	Notes
MT-ND2	223569.28496	6.714580	<1.000000e-300	MT Genes
MT-ATP8	19791.67322	6.220806	<1.000000e-300	MT Genes
MT-ND4	386421.87891	6.108083	3.046982e-245	MT Genes
MT-ND6	40817.58973	5.924393	3.221520e-157	MT Genes
MT-ND1	237368.08644	5.816280	1.000000e-300	MT Genes
MT-ND5	186848.25525	5.633187	2.989223e-110	MT Genes
MT-ATP6	160911.26793	5.271319	1.000000e-300	MT Genes
MT-ND4L	42080.11033	5.264596	1.000000e-300	MT Genes
MT-CYB	213642.50267	5.148185	2.808655e-153	MT Genes
MUC3A	440.12727	4.762839	4.963931e-18	
MT-CO2	257358.23841	3.374991	1.000000e-300	MT Genes
HSPB6	112.11844	3.171346	1.275501e-39	
NPIPB3	394.45526	3.035222	5.625690e-133	
ATN1	9071.84057	2.923674	9.395034e-74	
NPIPB13	24.24798	2.820896	7.263615e-15	
MT-ND3	45933.08320	2.778239	8.403991e-265	MT Genes
MT-CO1	593951.60914	2.775620	1.260887e-253	MT Genes
RSPO4	59.22689	2.742839	3.345901e-27	
IGF2	325.13770	2.674852	2.523008e-14	
KCNC3	1137.67484	2.628585	4.823953e-107	

Table S7. Number of cells per reference profile, real and simulated The "default" reference profile used only cells from Pool A and Pool B that were confidently assigned to a sample using genetic demultiplexing. The "hashing" used cells that were assigned to a sample by hash demultiplexing using Cell Ranger multi's default settings.

Cell type	Default	Hashing	Sim2000	Sim1000	Sim500	Sim200
B cells	323	214	43	22	11	4
Dendritic cells	249	135	33	16	8	3
Endothelial cells	814	631	109	55	27	11
Epithelial cells	1434	605	205	103	51	21
Fibroblasts	3336	1139	458	230	114	46
ILC	72	18	10	5	3	1
Macrophages	1348	837	181	91	45	18
Mast cells	39	24	6	3	1	1
Monocytes	454	289	62	31	16	6
NK cells	771	237	105	52	26	10
pDC	159	112	21	11	5	2
Plasma cells	759	335	107	54	27	11
T cells	4850	2998	664	331	165	66
Total	14608	7574	2004	1004	499	200

Table S8. Cell score deconvolution methods. All methods used are open source and return cell type scores, with a focus on immune cells.

Method	Implemented by	Uses scRNA-seq data	Availability
ABIS	Monaco et al 2019 [61]	No	R package
Consensus_TME	Jiménez-Sánchez et al 2019 [62]	No	R package
ImmuCellAI	Miao et al 2020 [63]	No	Web app
MCPcounter	Becht et al 2016 [64]	No	R package
quanTIseq	Finotello et al 2019 [65]	No	Bash script
TIMER	Li et al 2020 [66]	No	R package
XCell	Aran et al 2017 [67]	No	R package
