# Supplementary material for: Performance of computational algorithms to deconvolve heterogeneous bulk ovarian tumor tissue depends on experimental factors
Source: Genome Biol. 2023 Oct 20;24:239. doi: 10.1186/s13059-023-03077-7 (PMC10588129; doi:10.1186/s13059-023-03077-7)
Supplement: Supplementary file 2 — Additional file 2: Supplemental Figures 1–7. [file 13059_2023_3077_MOESM2_ESM.pdf]

Supporting Information for

Performance of computational algorithms to deconvolve heterogeneous bulk tumor tissue depends on experimental factors

Hippen, Omran, Weber, Jung, Drapkin, Doherty, Hicks, and Greene

Casey S. Greene, e-mail: casey.s.greene@cuanschutz.edu

This PDF file includes:

Figs S1 to S7

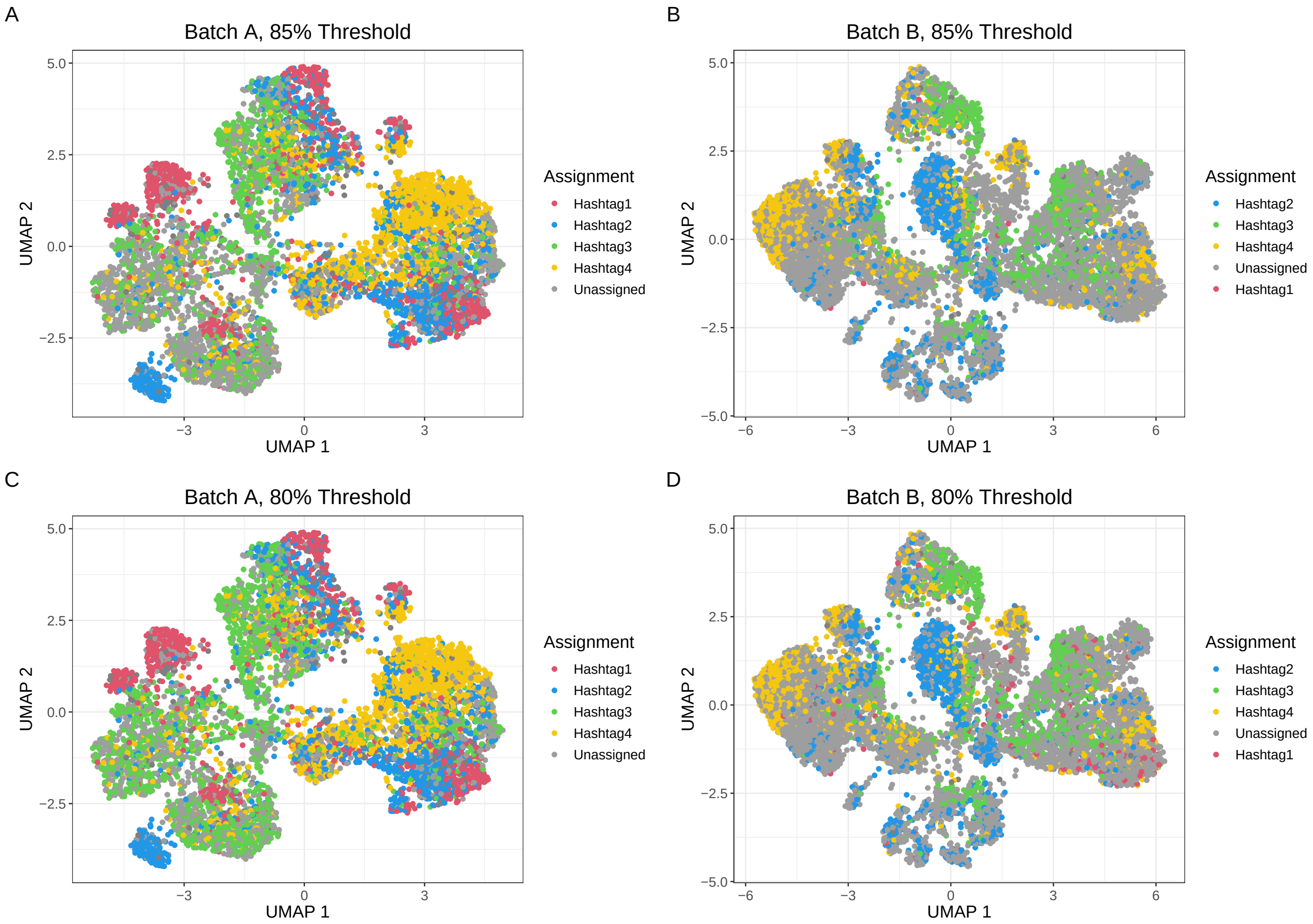

Fig. S1. Relaxed probability thresholds for hash demultiplexing increase number of assigned cells. A) Assignments for Batch A where any cell with greater than 85% probability of originating from a sample is assigned to that sample. B) Assignments for Batch B at the 85% probability threshold. C) Assignments for Batch A at a threshold of greater than 80% probability of originating from a sample. D) Assignments for Batch B at the 80% probability threshold.

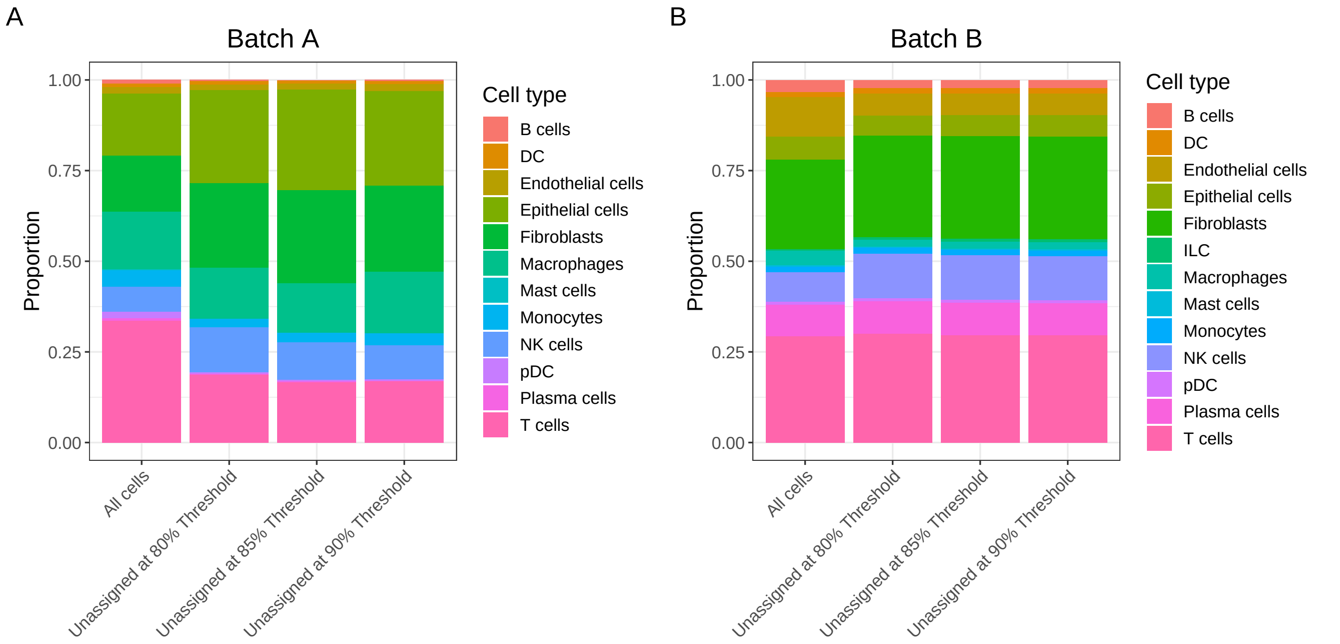

Fig. S2. Hash demultiplexing demonstrates cell type bias. A) Proportion of cell types in Batch A across all cells and in unassigned cells at various probability thresholds. Epithelial cells and fibroblasts are proportionally greater and T cells proportionally lesser in unassigned cells than in all cells. B) Proportion of cell types in Batch B.

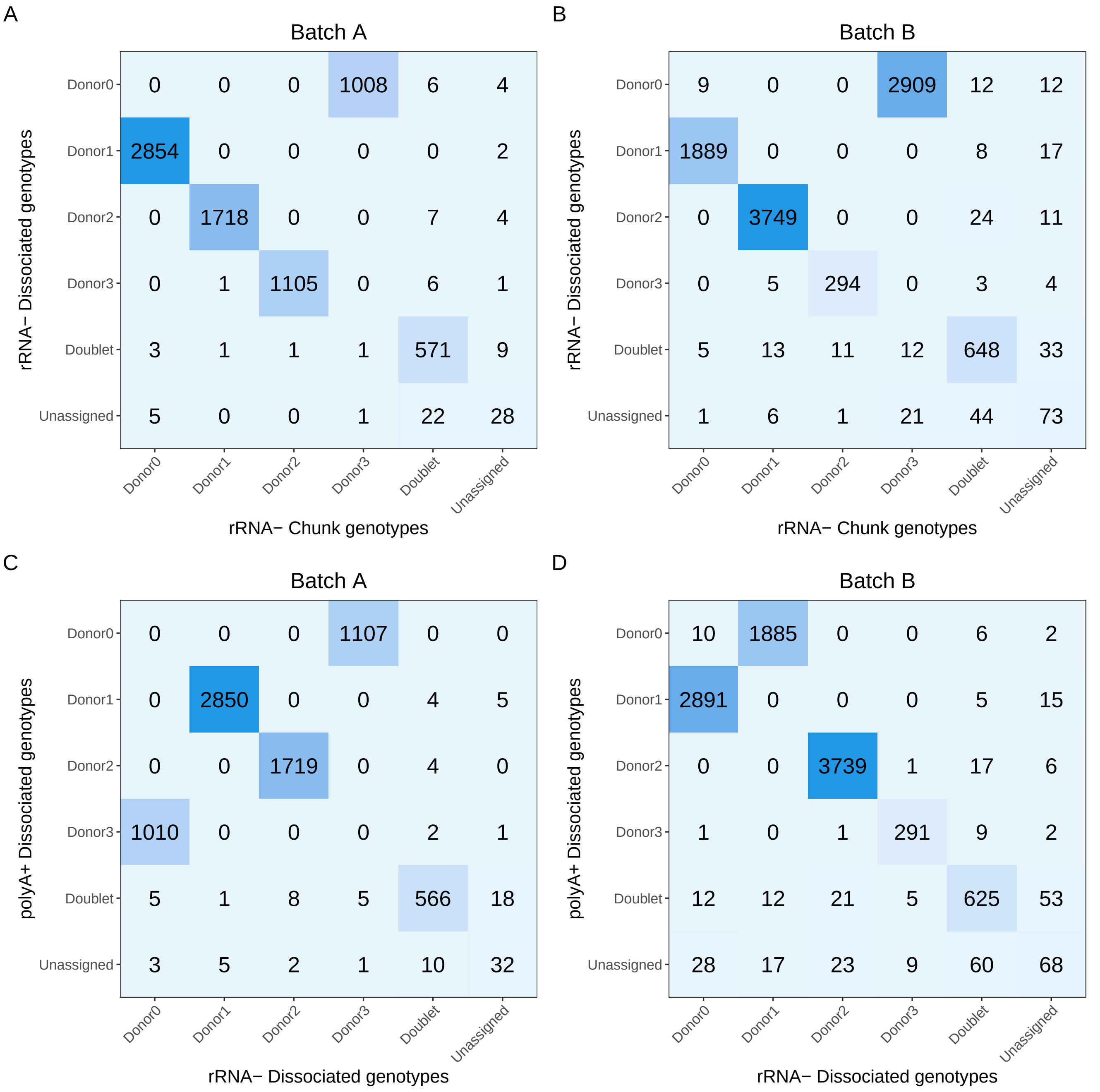

Fig. S3. Genetic demultiplexing is concordant across source of bulk reference genotypes. A) Confusion matrix of genetic demultiplexing assignments for Batch A when using reference genotypes from rRNA$^-$ Chunk samples vs rRNA$^-$ Dissociated samples. B) Genetic demultiplexing assignments for Batch B using reference genotypes from rRNA$^-$ Chunk samples vs rRNA$^-$ Dissociated samples. C) Confusion matrix of genetic demultiplexing assignments for Batch A when using reference genotypes from rRNA$^-$ Dissociated samples vs polyA$^+$ Dissociated samples. D) Genetic demultiplexing assignments for Batch B using reference genotypes from rRNA$^-$ Dissociated samples vs polyA$^+$ Dissociated samples.

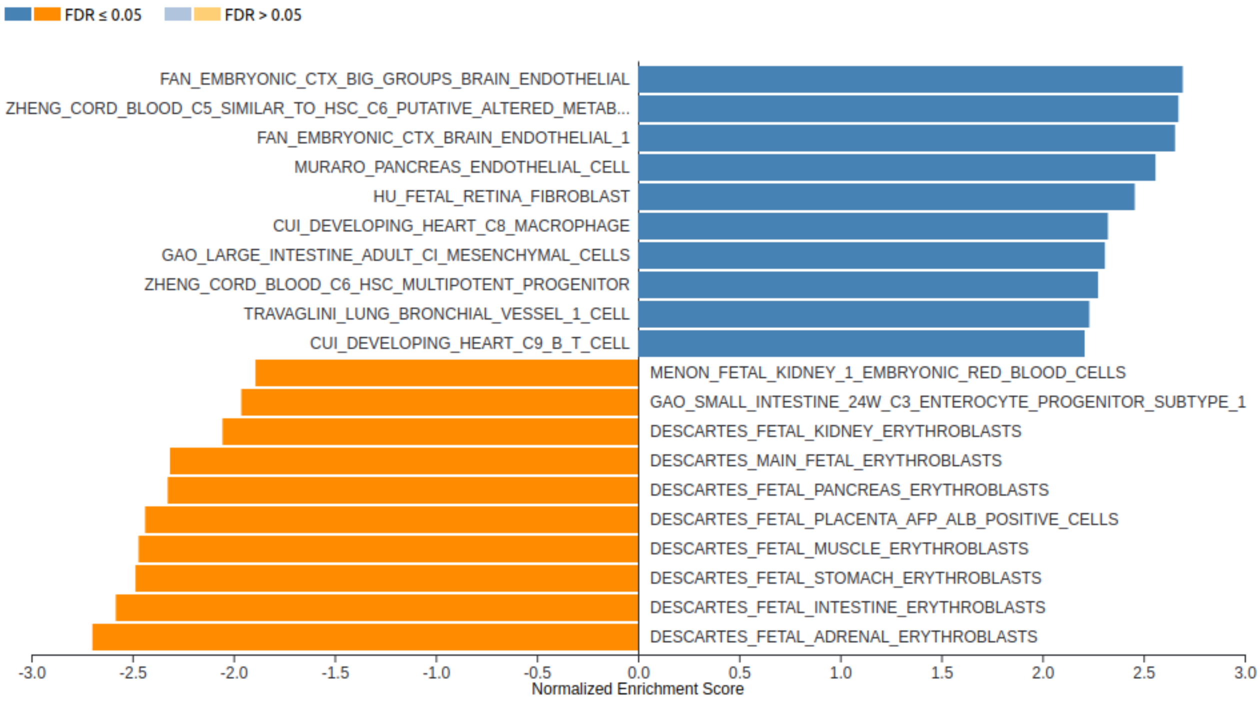

Fig. S4. Stromal cell types are more abundant in dissociated bulk samples. Results from Gene Set Enrichment Analysis of rRNA⁻ Chunk samples vs rRNA⁻ Dissociated samples. Gene signatures associated with endothelial cells, fibroblasts, macrophages, and other immune cells (blue) are more abundant in rRNA⁻ Dissociated samples, whereas red blood cell gene signatures (orange) are more abundant in rRNA⁻ Chunk samples.

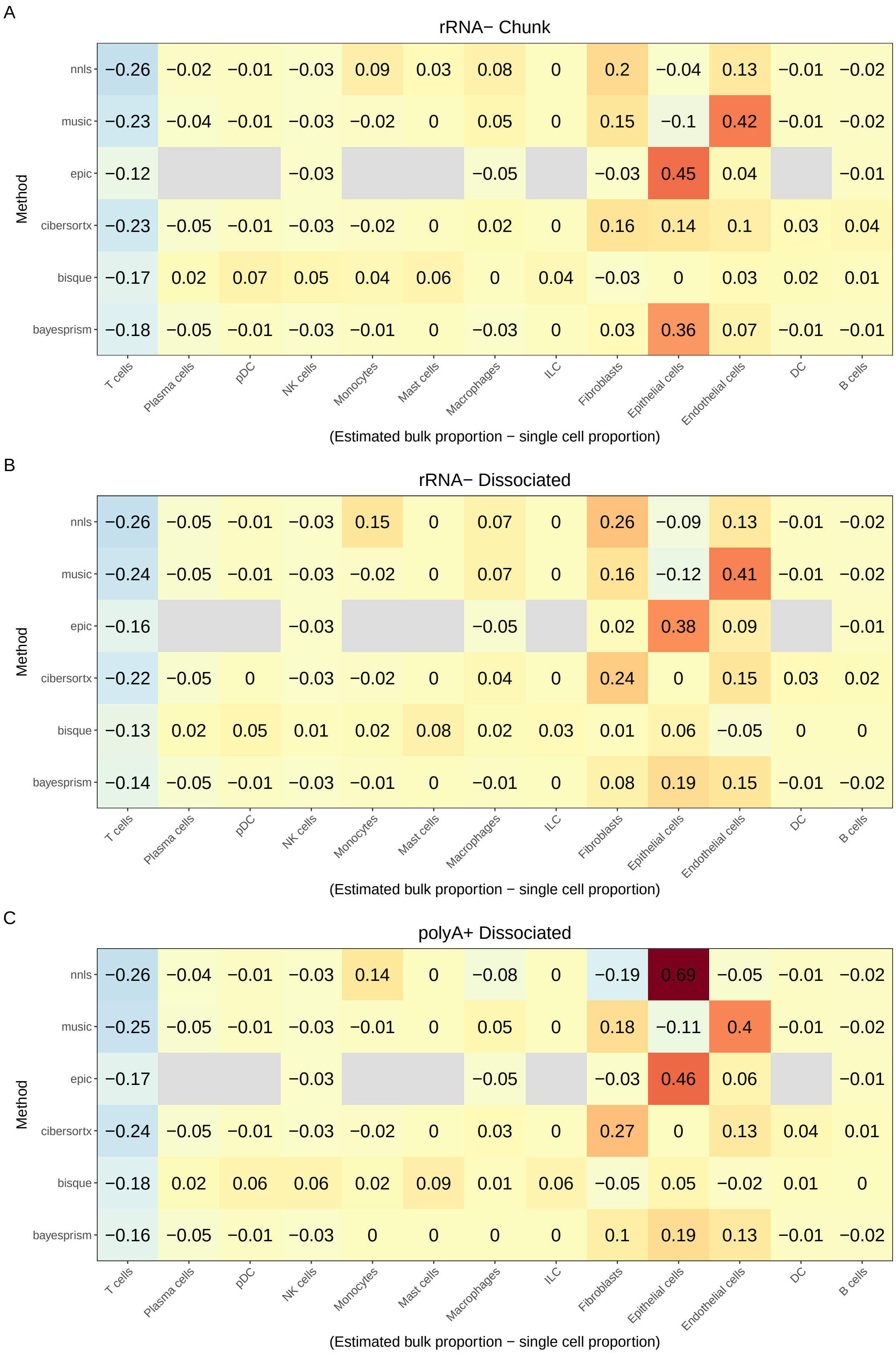

Fig. S5. Deconvolution estimates vary based on input bulk type. A) The average difference between estimated cell type proportion in rRNA$^-$ Chunk samples minus the corresponding cell type proportion in scRNA-seq Individual samples. Gray boxes represent cell types not estimated by a given method. B) The difference in cell type proportion based on rRNA$^-$ Dissociated sample deconvolution estimates and scRNA-seq Individual samples. C) The difference in cell type proportion based on polyA$^+$ Dissociated sample deconvolution estimates and scRNA-seq Individual samples.

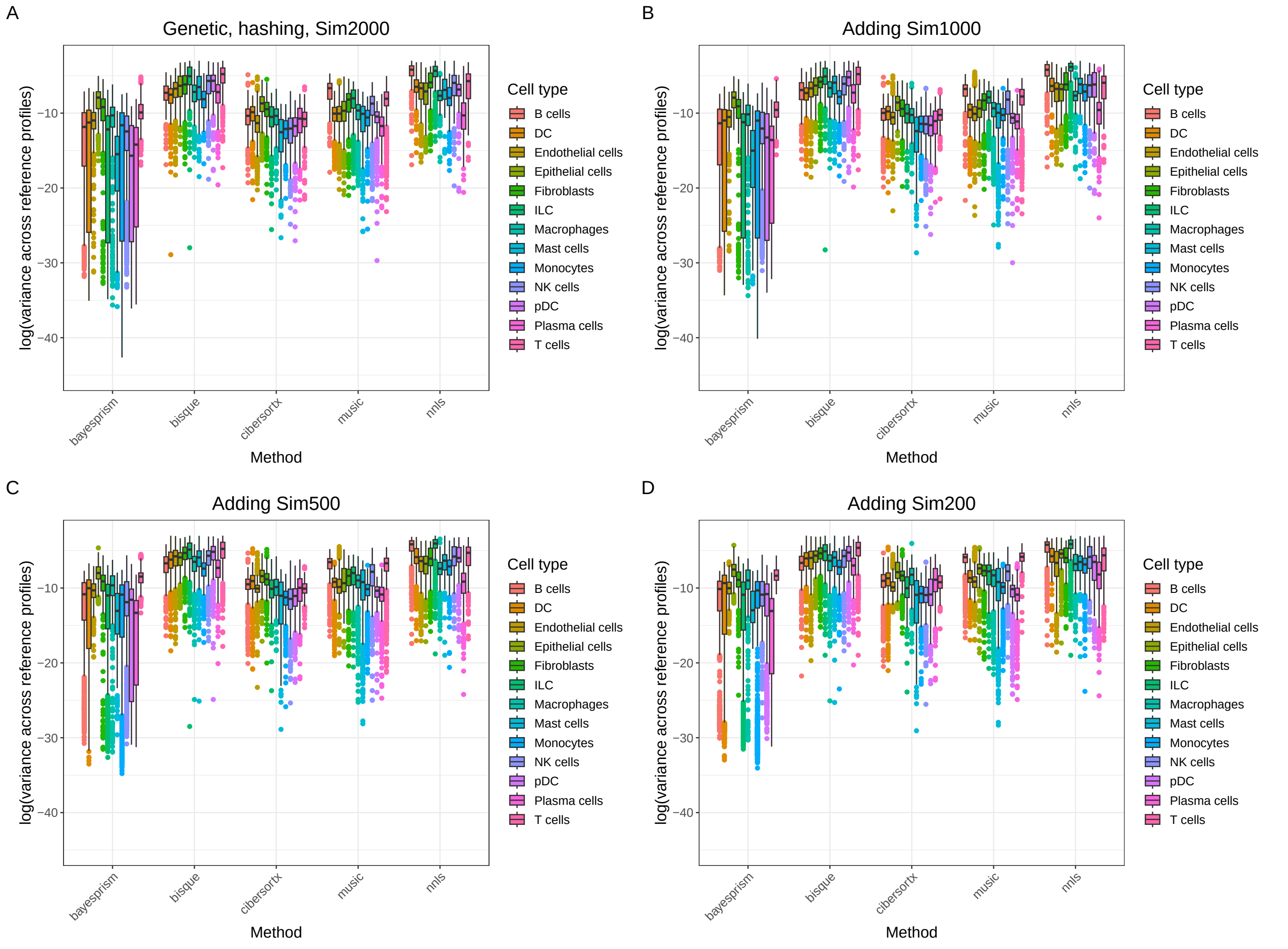

Fig. S6. Robustness to very small reference profiles A) The variance of deconvolution proportion estimates, stratified by cell type and method, when using our default reference profile (genetic), the reference profile of cells assigned to a sample by hash demultiplexing (hashing), and a simulated sample of approximately 2000 cells (Sim2000). B) Variance of proportion estimates across the same reference profiles as in A but also including results from Sim1000. C) Same as B but also including results from Sim500. D) Same as C but also including results from Sim200.

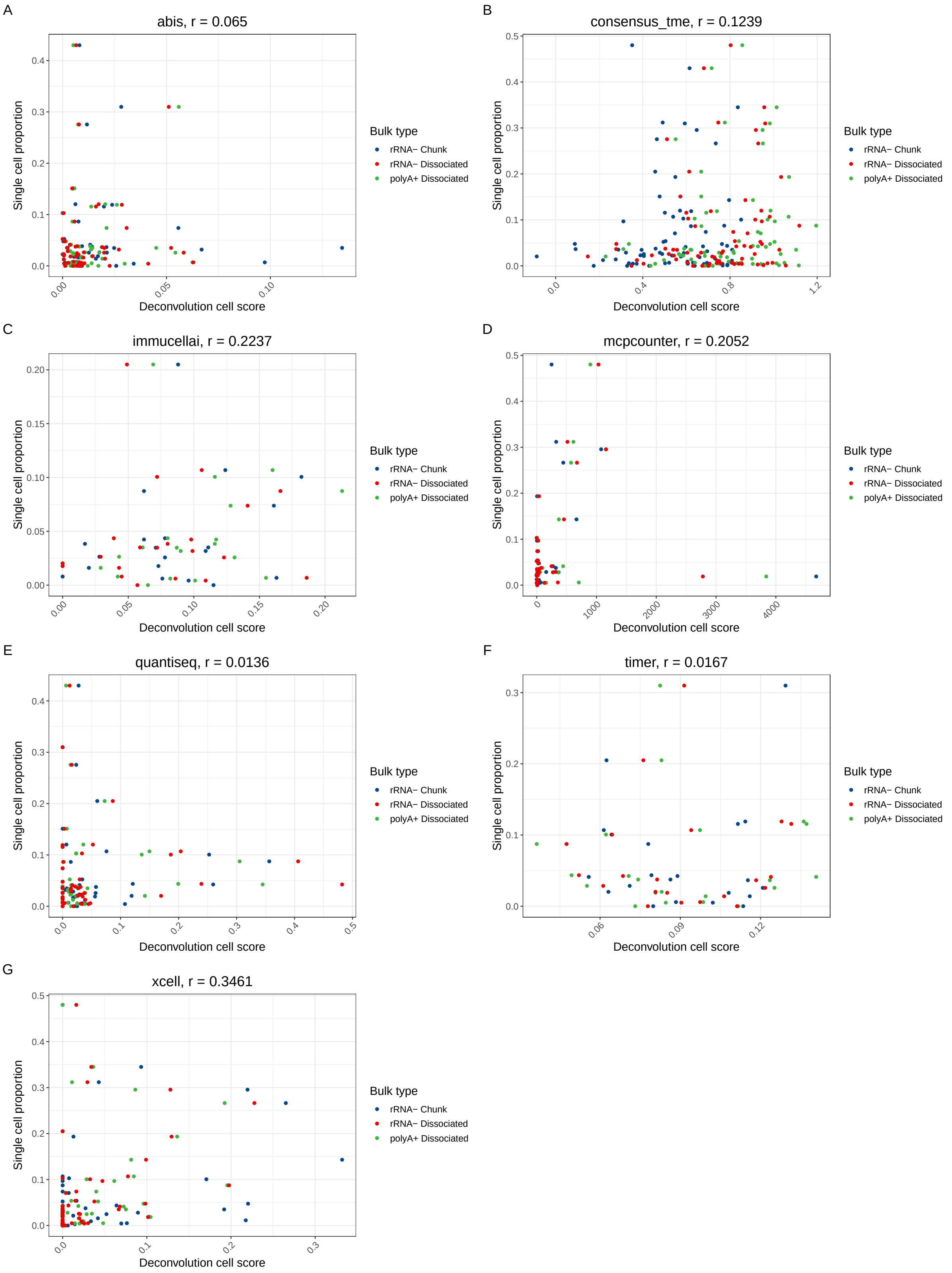

Fig. S7. Alternate deconvolution methods that return cell type scores do not match single cell proportions. A-G) Correlation between the cell type score returned by the deconvolution method and the corresponding proportion of cells in the scRNA-seq Individual sample. The name of the deconvolution method and the Pearson correlation (r value) is shown at the top of each panel.
