# Supplementary material for: Performance of computational algorithms to deconvolve heterogeneous bulk ovarian tumor tissue depends on experimental factors
Source: Genome Biol. 2023 Oct 20;24:239. doi: 10.1186/s13059-023-03077-7 (PMC10588129; doi:10.1186/s13059-023-03077-7)
Supplement: Supplementary file 3 — Additional file 3. Review history. [file 13059_2023_3077_MOESM3_ESM.docx]

**Review history**

**First round of review**

**Reviewer 1**

In this paper, Hippen et al. introduce a dataset and computational analysis to understand the important problem of how mismatch in transcriptional profiles between bulk and scRNA-seq affects the accuracy of cell type deconvolution algorithms. In particular, they consider the effects of cellular dissociation and varying ribosomal RNA removal methods (poly-A selection vs ribo-depletion) and generate paired scRNA-seq and bulk RNA-seq from a variety of protocols to answer this (e.g. pooled & demultiplexed vs separate, dissociate vs not, ribo-deplete vs poly-A select, etc) from primary high grade serous ovarian cancer tumours. They then perform a variety of informatic analysis to understand the effects of these choices, e.g. what genes are DE in bulk between dissociated vs not and similarly for poly-A vs ribo-deplete. They next apply a range of bulk deconvolution algorithms and compare the accuracy (inferred wrt pseudobulking and single cell data) for multiple methods, and quantify robustness to the choices of experimental protocol used to generate the underlying data.

Overall, this is an important paper that is valuable for the field. The particular strengths are (i) bringing to the attention of researchers issues caused by varying experimental choices on deconvolution accuracy, (ii) an important dataset that can be used for subsequent analysis, (iii) the computational analyses showing what cell types/pathways are impacted. However, I think the paper could be strengthened by additional edits and analyses that are detailed below, separated into major and minor.

Major

Paper flow: the introduction is really well written as an introduction to deconvolution / sample preparation. However, it doesn't really detail what's coming in the paper, what analysis is performed, what is found, etc. Can the authors expand on this? It would be really beneficial to add to figure 1 a summary table of the data (currently a separate table) as well as a schematic outlining the analyses to be performed. Also, titles throughout the paper could be more specific, e.g. "Genetic demultiplexing performs well in HGSOC samples" → Genetic-based approaches lead to higher cell demultiplexing rates in HGSOC samples. Finally, sometimes the text is confusing on a first read, e.g. "profiles revealed that individual sample types tended to segregate together" — "sample type" is really non descriptive, and it takes referencing the figure to realize this is patient/donor.

Use of correlation as a metric: in the deconvolution benchmark setup, the "even" setup performs poorly which is perhaps unexpected. One explanation would be that if all cell types have proportion roughly 1/C (for C cell types), and the deconvolution algorithm recovers the 1/C proportions near perfectly up to some small (random) noise, then the correlation between the true and predicted will be near zero (because correlation naturally scales each axis), but the absolute error in cell type predictions will be very low (i.e. very good predictions). Can the authors (i) plot out the true vs estimated proportions to see if this is the case, and (ii) repeat the analysis (e.g. figure 5B) using RSME (as has been done in some supplementary figures) to see if the same qualitative trend of simulation setup/algorithm performance holds as with the previous correlation-based analysis?

At various points it's claimed "Dissociation causes loss of certain cell types". However, this isn't exactly what's shown, rather that there's a reduction in marker genes associated with those cell types, which seems to be a good proxy for reduction in abundance of those cell types, but not strictly loss. Can the authors clarify whether they strictly mean loss, and if not update the manuscript to reflect a reduction?

A related issue is in the benchmarking of demultiplexing, the authors find a large number of cells are unassigned at default thresholds and hypothesize that this "could bias downstream deconvolution". However, this doesn't seem obvious from the results: are entire cell types missed from this, meaning they would be absent from the reference profiles? From Fig 6D the difference in proportions from multiplexed to normal seem minimal.

It would really strengthen the paper if the authors could comment on the relative impact of each of the steps considered (dissociate vs not, polyA vs ribo, demultiplex vs not, what deconvolution algorithm, etc) to the overall deconvolution estimates / variances, as it's not obvious what the relative importance of each step is. For example, should a practitioner take away that deconvolution algorithm choice is most important? Or polyA capture? Or something else? An ANOVA-style analysis could be used, or really anything that works.

I really think the title of the paper should make reference to Ovarian cancer to avoid the impression this is a pan-cancer analysis. While the dataset and analysis are really valuable, it is a limitation that only a single tumour type is examined so it is not necessarily straightforward how these findings will generalize. A sentence or two highlighting this in the discussion would also be beneficial.

Figure 3:

- Fig 3B it is not obvious that adipocyte genes are really preferentially on one side and a large number actually seem upregulated
- Fig 3B needs labelled as to which way is up (i.e. right is non-dissociated)
- The main text implies the only significant genes downregulated are HBB, but there are many other genes significantly up and down regulated beyond the erythrocyte/endothelial/adipocyte subsets shown. The paper would really benefit from a more detailed investigation into these, comparison with existing dissociation-associated gene sigs (e.g. [https://genomebiology.biomedcentral.com/articles/10.1186/s13059-019-1830-0](https://genomebiology.biomedcentral.com/articles/10.1186/s13059-019-1830-0)) and annotating e.g. top 20 up and down regulated genes on 3b regardless of cell type associated. This follows an emphasis in the paper on cell type proportion captures rather than the changing transcriptional state of the cells due to dissociation (even if cell type proportions are preserved) which would be beneficial to comment on
- It is written "We plotted other erythrocyte-specific genes [44] and found several were significantly more abundant in the tumor chunks as well." where is this figure?
- Figs 3C,D don't appear (from the axis title) normalized for read depth / library size etc.
- Same comments as above apply largely to figure 4 also, particularly figure labelling and investigation of other genes up/down regulated

Fig 5:

- Can authors check their cell type assignment? Fig 5A shows ~30% T cell and ~10% cellularity for epithelial cells which seems low for most epithelial tumours
- Figure 5C would be clearer if "true" was renamed to pseudobulk proportion
- Fig 5C, what simulation scenario is this? All?
- Figure titles need aligned better (e.g. figure 5B's x axis looks like the title of fig 5D)
- 5D is averaged over bulk preparation → can they split out by bulk preparation type?
- "Most of the methods trained on single-cell reference profiles performed best on the weighted simulations, whereas the methods that focus on immune cell types did poorly." → which methods are which? It would be helpful to reference and update Table 2 here

Fig 6:

- 6B has no commentary associated in the paper — the variation here seems low across everything with a large number of outliers. What is the conclusion of this analysis?
- The axes of figure 6 could be much more descriptive to improve readability, e.g. "bulk variance" → "variance in cell type proportion estimates in bulk samples"

Minor

- There are multiple mis-formatted references in the paper leading to [?]
- A short description of vireo when introduced would be beneficial
- Can the authors comment on why the cells are pooled into two batches for demultiplexing?

**Reviewer 2**

This manuscript by Hippen and colleagues presents a benchmarking study of computational deconvolution methods, using a novel dataset consisting of high grade serous ovarian tumors that have been profiled using both bulk RNA and scRNA-seq technologies, and also varied by experimental factors such as rRNA depletion vs polyA selection, demultiplexing strategy, etc. Unsurprisingly, they found dissociation protocols generally lead to bias in cell types sequenced, and that mRNA enrichment method selection also leads to changes in estimated gene abundances. They found deconvolution performance and robustness varied significantly depending on how they generated pseudo-bulk samples as well as depending on technical differences in how the data was generated.

Overall, I thoroughly enjoyed reading this manuscript and found it a nice departure from more typical deconvolution benchmarking studies (there is too much reliance on computational pseudo-bulk samples, one of the motivations of this paper). Highlights for me included:

- The introduction was exceptionally well written, and conveyed good motivation for the focus on tumor deconvolution (and the specific benefits of generating HGSOC-based benchmark data), and the need for real bulk samples (and associated single cell data) for deconvolution benchmarking as opposed to the more commonly used pseudo-bulk-based benchmarking.
- The design of the experiment (multiple bulk RNA and scRNA-seq based profiling strategies) is critical for understanding the impact of experimental choice on deconvolution performance, and also is something that obviously the common computational pseudo-bulk strategy cannot recapitulate. As far as I am aware, exploration of the specific effects of these experimental factors is also not as common in deconvolution benchmarks.

The main notable weakness in my mind was the lack of exploration into why there is a fairly large difference in how well specific cell types were predicted (e.g. T cells); more on that below.

Major comments:

- The authors point out and demonstrate that dissociation can lead to under-representation of specific cell types in the scRNA-seq data, such as adipocytes (or mesothelial cells in other tumor types), which may hamper deconvolution. But shouldn't this be addressable by using single cell reference data from more than just matched tumors (e.g. build a giant scRNA-seq or snRNA-seq atlas from multiple tissues, like perhaps the Human Cell Atlas or Tabula sapiens (https://tabula-sapiens-portal.ds.czbiohub.org/))? What prevents deconvolution methods from creating massively large reference data (from e.g. both matched tumors, as well as general healthy tissues which could have larger representation of those cell types that are more rare in the tumors?).

- Also, this raises a larger question (perhaps outside the direct scope of the paper but may be worth raising in the discussion), how many cells does one need of a particular cell type in a reference atlas in order to properly deconvolve/estimate the fraction of that cell type in a sample? The authors briefly touch on this point in the last results section where they look for consistency of each method across factors such as demultiplexing method (and where the number of cells in each cell type is presumably different across multiplexing methods), but it would be nice if the authors briefly did some experiments showing robustness specifically with respect to the number of cells each type is represented by in the single cell data.

- I find it would be very helpful if the authors varied feature selection for the scRNA-seq profiles and deconvolution experiments in general. For example, even when one does not strictly use only marker genes for deconvolution, deconvolution often improves with some light feature selection (e.g. HVGs). For Figure 4a, for example, if some light feature selection was performed on the scRNA-seq data to identify genes variable across cell types, then only those genes were used for PCA visualization, would the second PC still strongly distinguish rRNA- Dissociated and polyA+ Dissociated?

- I find it quite interesting that the deconvolution methods all performed most poorly on the even simulations (Fig. 5B). That seems counterintuitive, as I would have expected it to be most challenging to estimate the proportion of cell types that are more rare in the pseudo-bulk samples. I feel this may be the result of the performance evaluation metric here. Assuming that is Pearson correlation being measured, Pearson can achieve high values if the extreme values (smallest and largest abundances in this case) are predicted well. Is that the case here? It would be helpful if the authors commented on this. The authors state that the even scenario is the least realistic (which I agree), but what makes it a bit concerning is that does that suggest the tested deconvolution methods only work well if there are dominating cell types in the bulk samples? (e.g. can the methods perform well just by predicting essentially the largest component?)

- Have the authors checked whether there's a relationship between error in predicting a cell type's abundance and factors such as (1) # of cells in the single cell reference for that type; (2) relative abundance of that cell type in the mixture; or (3) heterogeneity of that cell type. I feel the results would be more interesting if the authors could delve into possible reasons for the variation in accuracy across cell types, as opposed to just making the observation.

- From the methods, it sounds like the pseudo-bulk construction procedure doesn't address differences in mRNA counts between cell types ('SimBu also offers a correction for different cell types having different amounts of mRNA, but we did not use this in our analysis in order to preserve the integer read counts.'). Does that mean the 'ground truth' cell type proportions is actually something more like the % cell contributions, and not % RNA contributions? This may affect accuracy estimates, as deconvolution methods typically predict % RNA contributions, not % cell contributions; can the authors compute the actual % RNA contributions and see if the per cell-type accuracies change materially?

Minor comments:
- The authors state "Also, HGSOC cells have high genomic instability and a particularly high burden of copy number variants [32, 33] which can complicate deconvolution." Presumably they mean HGSOC tumor cells would then be expected to have higher inter-patient and intra-patient variability (then leading to more difficult deconvolution)? Clarify.

- Figure 5C (and 5D) may be made more interpretable by scaling the y-axis by the abundance of the cell type. In other words, some cell types have higher abundances in the simulation than others; so larger errors for those more abundant cell types is less surprising (or egregious) compared to large errors for say, rare cell types.

**Authors’ response to reviewers**

The reviewers’ original comments are written in bold. Our point-by-point responses are written in normal text.

Reviewer #1: In this paper, Hippen et al. introduce a dataset and computational analysis to understand the important problem of how mismatch in transcriptional proﬁles between bulk and scRNA-seq affects the accuracy of cell type deconvolution algorithms. In particular, they consider the effects of cellular dissociation and varying ribosomal RNA removal methods (poly-A selection vs ribo-depletion) and generate paired scRNA-seq and bulk RNA-seq from a variety of protocols to answer this (e.g. pooled & demultiplexed vs separate, dissociate vs not, ribo-deplete vs poly-A select, etc) from primary high grade serous ovarian cancer tumours.

They then perform a variety of informatic analysis to understand the effects of these choices,

e.g. what genes are DE in bulk between dissociated vs not and similarly for poly-A vs ribo-deplete. They next apply a range of bulk deconvolution algorithms and compare the

accuracy (inferred wrt pseudobulking and single cell data) for multiple methods, and quantify robustness to the choices of experimental protocol used to generate the underlying data.

Overall, this is an important paper that is valuable for the ﬁeld. The particular strengths are (i) bringing to the attention of researchers issues caused by varying experimental choices on deconvolution accuracy, (ii) an important dataset that can be used for subsequent analysis,

(iii) the computational analyses showing what cell types/pathways are impacted. However, I think the paper could be strengthened by additional edits and analyses that are detailed below, separated into major and minor.

We appreciate the reviewer’s positive feedback. We agree that the additional edits and analyses strengthen the work, and we’ve responded point-by-point below to these suggestions.

Major

Paper ﬂow: the introduction is really well written as an introduction to deconvolution / sample preparation. However, it doesn't really detail what's coming in the paper, what analysis is performed, what is found, etc. Can the authors expand on this? It would be really beneﬁcial to add to ﬁgure 1 a summary table of the data (currently a separate table) as well as a schematic outlining the analyses to be performed. Also, titles throughout the paper could be more speciﬁc, e.g. "Genetic demultiplexing performs well in HGSOC samples" → Genetic-based approaches lead to higher cell demultiplexing rates in HGSOC samples. Finally, sometimes the text is confusing on a ﬁrst read, e.g. "proﬁles revealed that individual sample types tended to segregate together" — "sample type" is really non descriptive, and it takes referencing the ﬁgure to realize this is patient/donor.

We thank the reviewer for the suggestions they provided to improve the readability of the paper. We have updated figure 1 to include an overview of analyses we perform in the paper that directly compare one data type to another, with a note in the figure caption that all data types will be used for the comparison of deconvolution methods. We have reordered the introduction section such that the last paragraph is a clear outline of all analyses performed in the paper. We have also gone through the text and made changes for clarity, including making the section headings more specific as the reviewer suggested.

Use of correlation as a metric: in the deconvolution benchmark setup, the "even" setup performs poorly which is perhaps unexpected. One explanation would be that if all cell types have proportion roughly 1/C (for C cell types), and the deconvolution algorithm recovers the 1/C proportions near perfectly up to some small (random) noise, then the correlation between the true and predicted will be near zero (because correlation naturally scales each axis), but the absolute error in cell type predictions will be very low (i.e. very good predictions). Can the authors (i) plot out the true vs estimated proportions to see if this is the case, and (ii) repeat the analysis (e.g. ﬁgure 5B) using RSME (as has been done in some supplementary ﬁgures) to see if the same qualitative trend of simulation setup/algorithm performance holds as with the previous correlation-based analysis?

The reviewer’s intuition is correct: because all cell type proportions are approximately 1/C in the even simulation scenario, there is a smaller dynamic range on the true pseudo-bulk proportions and thus small errors in the estimated proportions can yield a Pearson correlation of near 0. We plotted the data and it appears that the range of estimated proportions is approximately comparable to the range of true proportions in the even simulation and the other simulation scenarios (see plots below).

Based on these data and comments from both reviewers, we have changed figures 5B, 6C, and 6E-F to use RMSE as a metric of accuracy instead of Pearson correlation.

At various points it's claimed "Dissociation causes loss of certain cell types". However, this isn't exactly what's shown, rather that there's a reduction in marker genes associated with those cell types, which seems to be a good proxy for reduction in abundance of those cell types, but not strictly loss. Can the authors clarify whether they strictly mean loss, and if not update the manuscript to reﬂect a reduction?

We thank the reviewer for this important distinction. We have amended the title of the subsection to say “Dissociation causes disproportionate loss of adipocyte and other cell type markers,” which is more directly supported by our bulk data results. We also added a note that while there isn’t complete loss of adipocyte markers in the dissociated bulk data, we did not ﬁnd any adipocytes in our paired scRNA-seq data. (It’s possible that the few adipocyte reads in the dissociated bulk data came from cellular debris that was strained prior to single-cell sequencing.) We are ultimately interested in how reference proﬁles made from scRNA-seq data affect deconvolution results, so the incomplete loss of adipocyte markers in bulk data is secondary to the seemingly more pronounced loss at the single cell level. To avoid confusion, however, we have changed all mentions of “loss” in the paper to “disproportionate loss”.

A related issue is in the benchmarking of demultiplexing, the authors ﬁnd a large number of cells are unassigned at default thresholds and hypothesize that this "could bias downstream deconvolution". However, this doesn't seem obvious from the results: are entire cell types missed from this, meaning they would be absent from the reference proﬁles? From Fig 6D the difference in proportions from multiplexed to normal seem minimal.

The reviewer is correct that this is unlikely to signiﬁcantly bias downstream deconvolution. The sentence they referenced was presented as a hypothetical outcome, but was not formally refuted after presentation of the data. We have added a sentence at the end of the paragraph clarifying that differential assignment of cells is unlikely to bias deconvolution except in extreme cases where entire cell types are missing.

It would really strengthen the paper if the authors could comment on the relative impact of each of the steps considered (dissociate vs not, polyA vs ribo, demultiplex vs not, what deconvolution algorithm, etc) to the overall deconvolution estimates / variances, as it's not obvious what the relative importance of each step is. For example, should a practitioner take away that deconvolution algorithm choice is most important? Or polyA capture? Or something else? An ANOVA-style analysis could be used, or really anything that works.

Thanks to the reviewer for this helpful suggestion on how to improve the paper. As they recommended, we performed an ANOVA to see how each technical factor affects the ﬁnal proportional estimate. We have added a subsection at the end of the results section describing our ﬁndings and have updated our experimental design recommendations in the discussion based on these ﬁndings.

I really think the title of the paper should make reference to Ovarian cancer to avoid the impression this is a pan-cancer analysis. While the dataset and analysis are really valuable, it is a limitation that only a single tumour type is examined so it is not necessarily

straightforward how these ﬁndings will generalize. A sentence or two highlighting this in the discussion would also be beneﬁcial.

We have altered the title to specify our analyses were performed on ovarian tumors, and have added a caveat to the discussion that results may vary for non-HGSOC tumors.

Figure 3:

- **Fig 3B it is not obvious that adipocyte genes are really preferentially on one side and a large number actually seem upregulated**

The adipocyte gene markers used in this paper are based on the clustering results from Emont et al 2022. We found that 70% of all adipose genes and 78% of the genes that exceeded traditional FDR and LFC thresholds are reduced in dissociation. In light of this and the other adipocyte-related results discussed earlier, we are conﬁdent moving forward with our characterization of adipocyte markers as reduced.

- **Fig 3B needs labelled as to which way is up (i.e. right is non-dissociated)**

We have added these labels to Figures 3 and 4.

- **The main text implies the only signiﬁcant genes downregulated are HBB, but there are many other genes signiﬁcantly up and down regulated beyond the erythrocyte/endothelial/adipocyte subsets shown. The paper would really beneﬁt from a more detailed investigation into these, comparison with existing dissociation-associated gene sigs (e.g. [**[**https://nam02.safelinks.protection.outlook.com/?url=https%3A%2F%2Fgenomebiology.biome**](https://genomebiology.biomedcentral.com/articles/10.1186/s13059-019-1830-0) [**dcentral.com%2Farticles%2F10.1186%2Fs13059-019-1830-0&data=05%7C01%7Ccasey.s.gre**](https://genomebiology.biomedcentral.com/articles/10.1186/s13059-019-1830-0) [**ene%40cuanschutz.edu%7C631fde9df25a4030163c08db36be228d%7C563337caa517421aaa**](https://genomebiology.biomedcentral.com/articles/10.1186/s13059-019-1830-0) [**e01aa5b414fd7f%7C0%7C0%7C638163962481768865%7CUnknown%7CTWFpbGZsb3d8eyJ**](https://genomebiology.biomedcentral.com/articles/10.1186/s13059-019-1830-0) [**WIjoiMC4wLjAwMDAiLCJQIjoiV2luMzIiLCJBTiI6Ik1haWwiLCJXVCI6Mn0%3D%7C3000%7C%7**](https://genomebiology.biomedcentral.com/articles/10.1186/s13059-019-1830-0)

[C%7C&sdata=iqJdXKWAqs0m0EP3vF%2FvvB%2BO2xjWkaEQHJoRGAQBHks%3D&reserved=0]](https://genomebiology.biomedcentral.com/articles/10.1186/s13059-019-1830-0) [(https://nam02.safelinks.protection.outlook.com/?url=https%3A%2F%2Fgenomebiology.biome](https://genomebiology.biomedcentral.com/articles/10.1186/s13059-019-1830-0) [dcentral.com%2Farticles%2F10.1186%2Fs13059-019-1830-0&data=05%7C01%7Ccasey.s.gre](https://genomebiology.biomedcentral.com/articles/10.1186/s13059-019-1830-0) [ene%40cuanschutz.edu%7C631fde9df25a4030163c08db36be228d%7C563337caa517421aaa](https://genomebiology.biomedcentral.com/articles/10.1186/s13059-019-1830-0) [e01aa5b414fd7f%7C0%7C0%7C638163962481768865%7CUnknown%7CTWFpbGZsb3d8eyJ](https://genomebiology.biomedcentral.com/articles/10.1186/s13059-019-1830-0) [WIjoiMC4wLjAwMDAiLCJQIjoiV2luMzIiLCJBTiI6Ik1haWwiLCJXVCI6Mn0%3D%7C3000%7C%7](https://genomebiology.biomedcentral.com/articles/10.1186/s13059-019-1830-0)

[C%7C&sdata=iqJdXKWAqs0m0EP3vF%2FvvB%2BO2xjWkaEQHJoRGAQBHks%3D&reserved=0](https://genomebiology.biomedcentral.com/articles/10.1186/s13059-019-1830-0)

)) and annotating e.g. top 20 up and down regulated genes on 3b regardless of cell type associated. This follows an emphasis in the paper on cell type proportion captures rather than the changing transcriptional state of the cells due to dissociation (even if cell type proportions are preserved) which would be beneﬁcial to comment on

We appreciate the reviewer identifying this important gap in our discussion of the differential expression results. We have now included the dissociation-related gene set from the O’Flanagan et al paper in our analysis and in Figure 3B. We have also added to the main text a discussion on how changing transcriptional state vs changes in cell type proportions may affect deconvolution. We found that including gene names in Figure 3B, even for only the top 20 or top

10 most up and downregulated genes, compromised the legibility of the ﬁgure, so we have instead included them as a supplemental table.

- **It is written "We plotted other erythrocyte-speciﬁc genes [44] and found several were signiﬁcantly more abundant in the tumor chunks as well." where is this ﬁgure?**

We have added a reference to the appropriate ﬁgure (3B) to this sentence.

- **Figs 3C,D don't appear (from the axis title) normalized for read depth / library size etc.**

Figures 3C and 3D are normalized based on size factor, as is the default for DESeq2, but the axis label did not specify this. We have changed the axis labels to “Normalized read counts.”

- **Same comments as above apply largely to ﬁgure 4 also, particularly ﬁgure labelling and investigation of other genes up/down regulated**

We applied the same changes as Figure 3 to Figure 4.

Fig 5:

- **Can authors check their cell type assignment? Fig 5A shows ~30% T cell and ~10% cellularity for epithelial cells which seems low for most epithelial tumours**

The reviewer is correct that these proportions don’t seem representative of the average epithelial tumor. Per their suggestion, we have reevaluated the cell type assignments based on canonical marker genes and are reasonably conﬁdent with our labels. The tumor depicted in Figure 5A is an extreme example that was used because it had the most observed cell types present, but we do see fewer epithelial cells and more T cells than we might otherwise expect across all of our single-cell samples. This supports the conclusion suggested by the data in Figure 5, that there may be additional cell type bias introduced by single-cell sequencing that oversamples T cells and undersamples ﬁbroblasts and epithelial cells in scRNA-seq data.

- **Figure 5C would be clearer if "true" was renamed to pseudobulk proportion**
- **Fig 5C, what simulation scenario is this? All?**

We have clariﬁed in the ﬁgure caption that these results are aggregated across all simulation scenarios.

- **Figure titles need aligned better (e.g. ﬁgure 5B's x axis looks like the title of ﬁg 5D)**

We have reformatted the ﬁgure and the axis labels should be better aligned.

- **5D is averaged over bulk preparation → can they split out by bulk preparation type?**

Supplemental ﬁgure 5 is intended to show that the conclusions of ﬁgure 5D are similar across bulk preparation type. We examined stratiﬁed bar plots, but ultimately found that the heatmap of means in ﬁgure S5 was more directly comparable across bulk types.

- **"Most of the methods trained on single-cell reference proﬁles performed best on the weighted simulations, whereas the methods that focus on immune cell types did poorly." → which methods are which? It would be helpful to reference and update Table 2 here**

This sentence was written before we decided that methods that technically give proportions but focus much more on immune types were better examined in the supplemental figure with the immune-specific cell type “score” methods. The whole section has been updated

to account for the change from Pearson correlation to RMSE, so this confusing language has been removed.

Fig 6:

- **6B has no commentary associated in the paper — the variation here seems low across everything with a large number of outliers. What is the conclusion of this analysis?**

Based on this comment and suggestions from Reviewer 2, we have signiﬁcantly expanded this section to comment on how the size of reference proﬁles affects deconvolution method robustness using simulated small reference proﬁles.

- **The axes of ﬁgure 6 could be much more descriptive to improve readability, e.g. "bulk variance" → "variance in cell type proportion estimates in bulk samples"**

We have changed the axis labels of ﬁgure 6 to be clearer and more consistent with the axis labels of ﬁgure 5.

Minor

- **There are multiple mis-formatted references in the paper leading to [?]**

The mis-formatted references have now been corrected.

- **A short description of vireo when introduced would be beneﬁcial**

We have added language about how vireo assigns cells to samples when it is ﬁrst introduced in the paper.

- **Can the authors comment on why the cells are pooled into two batches for demultiplexing?**

The primary reason not to do one pool of eight samples was to avoid loading too many cells at once and increasing the risk of a clog in the cell strainer. We have now specified this as a reason in the results and methods sections.

Reviewer #2: This manuscript by Hippen and colleagues presents a benchmarking study of computational deconvolution methods, using a novel dataset consisting of high grade serous ovarian tumors that have been proﬁled using both bulk RNA and scRNA-seq technologies, and also varied by experimental factors such as rRNA depletion vs polyA selection, demultiplexing strategy, etc. Unsurprisingly, they found dissociation protocols generally lead to bias in cell types sequenced, and that mRNA enrichment method selection also leads to changes in estimated gene abundances. They found deconvolution performance and robustness varied signiﬁcantly depending on how they generated pseudo-bulk samples as well as depending on technical differences in how the data was generated.

Overall, I thoroughly enjoyed reading this manuscript and found it a nice departure from more typical deconvolution benchmarking studies (there is too much reliance on computational pseudo-bulk samples, one of the motivations of this paper). Highlights for me included:

- **The introduction was exceptionally well written, and conveyed good motivation for the focus on tumor deconvolution (and the speciﬁc beneﬁts of generating HGSOC-based benchmark data), and the need for real bulk samples (and associated single cell data) for deconvolution benchmarking as opposed to the more commonly used pseudo-bulk-based benchmarking.**
- **The design of the experiment (multiple bulk RNA and scRNA-seq based proﬁling strategies) is critical for understanding the impact of experimental choice on deconvolution performance, and also is something that obviously the common computational pseudo-bulk strategy cannot recapitulate. As far as I am aware, exploration of the speciﬁc effects of these experimental factors is also not as common in deconvolution benchmarks.**

The main notable weakness in my mind was the lack of exploration into why there is a fairly large difference in how well speciﬁc cell types were predicted (e.g. T cells); more on that below.

We appreciate the reviewer’s positive feedback on the work. We sought, with the introduction, to provide important contextualization, and we are happy that it was exceptional. We have now performed the analyses suggested by the reviewer, which we feel strengthened the manuscript.

Major comments:

- **The authors point out and demonstrate that dissociation can lead to**

under-representation of speciﬁc cell types in the scRNA-seq data, such as adipocytes (or mesothelial cells in other tumor types), which may hamper deconvolution. But shouldn't this be addressable by using single cell reference data from more than just matched tumors (e.g. build a giant scRNA-seq or snRNA-seq atlas from multiple tissues, like perhaps the Human Cell Atlas or Tabula sapiens ([https://nam02.safelinks.protection.outlook.com/?url=https%3A%2F%2Ftabula-sapiens-portal.](https://tabula-sapiens-portal.ds.czbiohub.org/) [ds.czbiohub.org%2F&data=05%7C01%7Ccasey.s.greene%40cuanschutz.edu%7C631fde9df25](https://tabula-sapiens-portal.ds.czbiohub.org/) [a4030163c08db36be228d%7C563337caa517421aaae01aa5b414fd7f%7C0%7C0%7C6381639](https://tabula-sapiens-portal.ds.czbiohub.org/)

[62481768865%7CUnknown%7CTWFpbGZsb3d8eyJWIjoiMC4wLjAwMDAiLCJQIjoiV2luMzIiLCJ](https://tabula-sapiens-portal.ds.czbiohub.org/) [BTiI6Ik1haWwiLCJXVCI6Mn0%3D%7C3000%7C%7C%7C&sdata=2G1mS0Kp9A7%2FGYd2k9a](https://tabula-sapiens-portal.ds.czbiohub.org/)

[WUIBgvtuXuzyLKo7JAcxG0HI%3D&reserved=0](https://tabula-sapiens-portal.ds.czbiohub.org/)))? What prevents deconvolution methods from creating massively large reference data (from e.g. both matched tumors, as well as general healthy tissues which could have larger representation of those cell types that are more rare in the tumors?).

The reviewer has touched upon an interesting avenue for discovery. We agree that, in theory, a large atlas could provide the full set of cell types necessary for any deconvolution. However, there are some challenges with that approach: individual cell types can have different phenotypic and transcriptional proﬁles in different tissue contexts, and the precise differences are not always well characterized. While we feel this is an important research question, we hesitate to recommend combining cell data from other tissues into a deconvolution reference proﬁle, even matched healthy tissues for tumor deconvolution, before the difference in tissue contexts is better understood. It is possible, as the reviewer suggests, that a suﬃciently large

and diverse reference dataset would allow deconvolution methods to learn unifying features of the reference proﬁle cell types and perform well. Though we consider it outside the scope of this paper, we look forward to seeing this evaluated experimentally in future work. We have added a sentence to the Discussion to note this possibility.

- **Also, this raises a larger question (perhaps outside the direct scope of the paper but may be worth raising in the discussion), how many cells does one need of a particular cell type in a reference atlas in order to properly deconvolve/estimate the fraction of that cell type in a sample? The authors brieﬂy touch on this point in the last results section where they look for consistency of each method across factors such as demultiplexing method (and where the number of cells in each cell type is presumably different across multiplexing methods), but it would be nice if the authors brieﬂy did some experiments showing robustness speciﬁcally with respect to the number of cells each type is represented by in the single cell data.**

We thank the reviewer for this suggestion, which we believe improves the value of the other robustness analyses done in the paper. We have performed a “stress test” of sorts, seeing how the output of deconvolution changes with smaller and smaller simulated reference proﬁles (with 2000, 1000, 500, and 200 total cells). We found that methods that had already proved to be robust were able to perform decent quantiﬁcation of cell types with as few as 3 cells in the reference proﬁle. We have added a supplemental ﬁgure demonstrating the effect of small reference proﬁles on variance of deconvolution estimates across cell type and method. We have also added a paragraph on our ﬁndings in the results section and details about our simulated reference proﬁles in the methods section.

- **I ﬁnd it would be very helpful if the authors varied feature selection for the scRNA-seq proﬁles and deconvolution experiments in general. For example, even when one does not strictly use only marker genes for deconvolution, deconvolution often improves with some light feature selection (e.g. HVGs). For Figure 4a, for example, if some light feature selection was performed on the scRNA-seq data to identify genes variable across cell types, then only those genes were used for PCA visualization, would the second PC still strongly distinguish rRNA- Dissociated and polyA+ Dissociated?**

The reviewer’s intuition is absolutely correct that feature selection can affect many of the results we describe in the paper. Speciﬁcally in the case of Figure 4A, as the reviewer notes, the second principal component strongly segregated the rRNA- Dissociated and polyA+ Dissociated samples (original ﬁgure with no feature selection and 36,000 genes included below for reference):

As the reviewer recommended, we performed light feature selection on our single-cell data, selecting HVGs that had a greater than zero variance attributed to biological differences and not technical noise (approximately 11,000 genes total). When we ﬁltered our bulk results to these genes and performed PCA, we found the segregation on PC2 to be very similar (see below):

More stringent feature selection of only using the top 20% most highly variable genes in the single-cell data (approximately 2,400 genes) showed the rRNA- dissociated and polyA+ dissociated samples starting to come together on PC2, but only just:

It is possible that with extremely stringent feature selection, PC2 would no longer distinguish samples based on mRNA enrichment method, but the relevance of such an analysis diminishes when it’s being performed on a very small gene set. Because the interpretation of the results is the same for no feature selection and for light feature selection, we have opted to keep the original figure 4A as is. We did not perform feature selection on the deconvolution input data, as each deconvolution method has its own criteria for identifying relevant features and filtering features with low variance. To keep the comparison of the methods consistent, we gave all genes as input for deconvolution except where recommended by the authors of the given method, e.g. restricting BayesPrism analysis to protein-coding genes.

- **I ﬁnd it quite interesting that the deconvolution methods all performed most poorly on the even simulations (Fig. 5B). That seems counterintuitive, as I would have expected it to be most challenging to estimate the proportion of cell types that are more rare in the pseudo-bulk samples. I feel this may be the result of the performance evaluation metric here. Assuming that is Pearson correlation being measured, Pearson can achieve high values if the extreme values (smallest and largest abundances in this case) are predicted well. Is that the case here? It would be helpful if the authors commented on this. The authors state that the even scenario is the least realistic (which I agree), but what makes it a bit concerning is that does that suggest the tested deconvolution methods only work well if there are dominating cell types in**

the bulk samples? (e.g. can the methods perform well just by predicting essentially the largest component?)

We agree with the reviewer that the results of 5B indicate that Pearson correlation is not the best metric to assess accuracy here. We have changed 5B to use the reduced mean squared error (RMSE), which should be less biased by having one dominating cell type than Pearson correlation.

- **Have the authors checked whether there's a relationship between error in predicting a cell type's abundance and factors such as (1) # of cells in the single cell reference for that type; (2) relative abundance of that cell type in the mixture; or (3) heterogeneity of that cell type. I feel the results would be more interesting if the authors could delve into possible reasons for the variation in accuracy across cell types, as opposed to just making the observation.**

We evaluated the impact of the number of cells in the single cell reference using the “stress test” described above. We found that some methods had built-in preventions from quantifying extremely rare cell types. And by downsampling for the stress test but requiring at least one cell per cell type, we also could evaluate how altering the relative proportions of cell types in the reference affects their estimations in the mixture. We found that in several cases with an extremely downsampled reference (sim200), the proportion that was otherwise assigned to macrophages and T cells were assigned to more rare immune cell types. And as we expected, the more abundant cell types in the mixture had higher variance, since more variance is possible for higher values with a non-negativity constraint.

Based on the results of the stress test, we believe that heterogeneity of a cell type likely does impact the performance of a deconvolution method in quantifying that cell type. For instance, in our best-performing methods, the highly heterogeneous cell types of cancer epithelial cells and T cells had the highest variance when using smaller reference profiles. Given the limitations of our dataset (we did not perform FACS on our data or other experimental techniques that would allow us to identify cell types and states more granularly), we feel that a full exploration of the effect of cell type heterogeneity on deconvolution would be better addressed in future work.

- **From the methods, it sounds like the pseudo-bulk construction procedure doesn't address differences in mRNA counts between cell types ('SimBu also offers a correction for different cell types having different amounts of mRNA, but we did not use this in our analysis in order to preserve the integer read counts.'). Does that mean the 'ground truth' cell type proportions is actually something more like the % cell contributions, and not % RNA contributions? This may affect accuracy estimates, as deconvolution methods typically predict**

% RNA contributions, not % cell contributions; can the authors compute the actual % RNA contributions and see if the per cell-type accuracies change materially?

We thank the reviewer for encouraging us to make the distinction between cell abundance and mRNA abundance. We reran the analyses in Figure 5B-D using % RNA contributions as a metric instead of cell contributions. We sampled the cells based on the parameters described in the paper using SimBu, but then calculated the number of reads

contributed by each cell type’s sampled cells, and used that as the pseudo-bulk true proportion instead of the proportion of total cells. When we did this, we found the cell type bias in

pseudo-bulk accuracy was attenuated. We have changed the corresponding results section to reﬂect these changes. We found that the cell type biases in the real bulk data were also smaller, but still present. From this, we conclude that some of the results we found in the ﬁrst version of the paper were due to differences in mRNA counts across cell types–but not all. This strengthens our conclusion that focusing on only pseudo-bulk accuracy when assessing a deconvolution method is insuﬃcient. We have updated ﬁgure 5 and ﬁgure 6E as well as the methods section to reﬂect the change in calculating pseudo-bulk accuracy.

Minor comments:

- **The authors state "Also, HGSOC cells have high genomic instability and a particularly high burden of copy number variants [32, 33] which can complicate deconvolution." Presumably they mean HGSOC tumor cells would then be expected to have higher inter-patient and intra-patient variability (then leading to more diﬃcult deconvolution)? Clarify.**

We have added a sentence to clarify that changes in copy number alter the baseline gene expression of cancer cells in a patient-speciﬁc way, increasing inter-patient heterogeneity.

- **Figure 5C (and 5D) may be made more interpretable by scaling the y-axis by the abundance of the cell type. In other words, some cell types have higher abundances in the simulation than others; so larger errors for those more abundant cell types is less surprising (or egregious) compared to large errors for say, rare cell types.**

The reviewer is correct that more abundant cell types will naturally have larger errors. We considered several different ways to scale the y axis of ﬁgures 5C and 5D, but found that none produced a more visually interpretable result. Scaling by abundance, whether by the pseudo-bulk proportion of the individual or by aggregated cell type abundance across all samples, meant that the errors for rare cell types were divided by very small coeﬃcients, forming extreme outliers that compromised the plot’s legibility (see ﬁgure below). Because of this, we instead opted to note in the text body that we would expect errors in the more abundant cell types to be bigger. The consistent directionality of the errors in the abundant cell types is what leads us to conclude there may be cell type bias introduced by single-cell sequencing.

**Second round of review**

**Reviewer 1**

The authors have addressed all my concerns and I think this is a very strong paper worthy of publication.

**Reviewer 2**

The authors have fully addressed my comments with this revision.
